# Dual-Confinement Strategy Enables Highly Efficient Oxygen Reduction with Fe–N_5_ Electrocatalysts

**DOI:** 10.34133/research.1333

**Published:** 2026-06-17

**Authors:** Shilei Li, Jingshuo Liu, Zhihang Liu, Congcong Yang, Jian Li, Zhiqun Lin, Likun Gao

**Affiliations:** ^1^ State Key Laboratory of Woody Oil Resources Utilization, Northeast Forestry University, Harbin 150040, PR China.; ^2^ Key Laboratory of Bio-based Material Science & Technology, Ministry of Education, Northeast Forestry University, Harbin 150040, PR China.; ^3^ Department of Chemical and Biomolecular Engineering, National University of Singapore, 117585 Singapore.

## Abstract

Fe single-atom catalysts with well-defined porous architectures and optimized Fe–N*_x_* microenvironments show great potential for enhancing the oxygen reduction reaction. Herein, we report Fe–N_5_ single-atom catalysts, where each Fe atom coordinates with 4 pyridinic N and 1 axial pyrrolic N, for efficient oxygen reduction. A dual-confinement strategy, combining the wood framework with Fe^3+^ coordination, directs self-assembly of cellulose nanocrystals (CNCs) into a porous wood-derived architecture. Subsequent pyrolysis yields Fe–N_5_ catalysts anchored on N, S-codoped carbon with hollow, hierarchically interconnected 3-dimensional pores. Notably, coordination between CNCs and Fe^3+^ guides the formation of Fe–N*_x_* moieties within a tailored microenvironment, enabling control over the coordination number, heteroatom doping, and the electronic structure. X-ray absorption spectroscopy and density functional theory calculations reveal that FeN_5_ moieties are optimized through 3 synergistic factors: Fe coordination with 4 pyridinic N and 1 axial pyrrolic N, S doping from residual sulfate ester groups in CNCs, and adjacent micropores. Collectively, these effects lower the *OH desorption barrier, accelerating the adsorption/desorption of oxygenated intermediates. Consequently, Fe–N_5_ single-atom catalysts exhibit an exceptional oxygen reduction reaction activity with a half-wave potential of 0.964 V. This dual-confinement strategy enables high-performance non-precious-metal catalysts for metal–air batteries, as evidenced by Fe–N_5_-based zinc–air batteries outperforming Pt/C.

## Introduction

Single-atom catalysts (SACs), particularly Fe single atoms anchored on N-doped carbon support (denoted Fe–N–C), have emerged as highly promising non-precious-metal electrocatalysts for the oxygen reduction reaction (ORR) in clean energy systems, including fuel cells and zinc–air batteries (ZABs). They offer a sustainable alternative to platinum-based catalysts, which are limited by scarcity and high cost [1,2]. Hierarchical porous structures with hollow-interconnected channels are widely recognized as an effective design for highly active Fe–N–C catalysts, as they maximize the accessibility of active sites [3]. The presence of micropores enhances the specific surface area, creating more exposed active sites; in contrast, mesopores and macropores accelerate the transport of oxygen, reaction intermediates, and water molecules [3,4]. Conventional synthesis approaches for Fe-based SACs primarily involve confined pyrolysis of metal–organic frameworks or direct coordination between metal ions and polymeric ligands [1]. These methods often require high-cost reagents, organic solvents, and complex precursor preparation, resulting in poor economic feasibility, incomplete hierarchical structures, and high propensity for metal agglomeration during carbonization [5]. Furthermore, enhancing ORR performance typically necessitates additional heteroatom doping (e.g., S, N, and P), which further increases synthetic complexity and impairs experimental reproducibility [2]. Thus, it remains an urgent need for exploring cost-effective and controllable strategies to fabricate Fe-based SACs with asymmetric coordination and high atomic utilization, despite the technical challenges involved.

Cellulose nanocrystals (CNCs), renewable nanomaterials derived from natural sources, have been extensively used as support for metal-based materials due to their large specific surface area and rich surface functional moieties [6]. These surface groups, including –OH, –COOH, and –OSO_3_H, enable coordination with metal ions, promoting the dispersion and stabilization of Fe^3+^ ions [7]. To further enhance the metal–ion coordination capability of CNCs, carboxylation treatment is commonly employed to oxidize –OH groups into –COOH groups [8,9]. The self-assembly of CNCs, mediated by coordination confinement [10,11] or spatial confinement [12], has been utilized to engineer porous nanoarchitectures [13]. As multidentate ligands, Fe^3+^ ions can bridge multiple CNCs via coordination confinement, driving self-assembly of CNCs into ordered 2-dimensional or 3-dimensional (3D) networks and markedly improving structural stability [14]. Coordination confinement arises from electrostatic adsorption and coordination interactions between CNC surface groups (–OH, –COOH, and –OSO_3_H) and Fe^3+^ ions, forming C–O···Fe^3+^ bonds under acidic conditions [15,16]. Moreover, the spatial distribution of Fe^3+^ ions enables in situ generation of atomic-level dispersed Fe active centers embedded in carbon-based carriers after thermal treatment [17,18]. On the other hand, self-assembly of CNCs can also be guided by geometric boundaries, resulting in distinct structural features that profoundly modulate material properties [19]. Additionally, regulating the morphology of the carbon matrix can alter the coordination microenvironment of SACs, which critically affects catalytic activity and stability [3]. For instance, compared with the 4-coordinated Fe–N_4_ moiety, the 5-coordinated Fe–N_5_ structure lowers the reaction activation energy and improves catalytic kinetics due to its favorable electronic configuration and dynamic coordination behavior [8]. However, how to modulate the local microenvironment of Fe–N_5_ sites through heteroatom doping and axial N coordination while simultaneously enhancing mass transfer efficiency and exposing more active sites to break the limitation of their intrinsic activity remains a formidable challenge.

Herein, we report a dual-confinement strategy that integrates the wood framework and Fe^3+^ ion coordination to assemble 2,2,6,6-tetramethylpiperidin-1-oxyl (TEMPO)-modified CNCs (T-CNCs) into porous wood-derived architecture, yielding Fe–N_5_ SACs with hierarchical porosity after pyrolysis. Notably, –OH groups on the wood cell wall form hydrogen bonds with T-CNCs, enhancing their structural stability. Each Fe atom in Fe–N_5_ SACs is bonded to 4 basal pyridinic N atoms and a single axial pyrrolic N, enabling efficient ORR for ZABs. X-ray absorption spectroscopy (XAS) and first-principles calculations revealed that the electronic configuration of Fe sites is modulated by 3 factors: axial pyrrolic N with 4 basal pyridinic N in Fe–N_5_, in situ S doping from T-CNCs, and abundant hole defects. These synergistic effects reduce the reaction energy barrier, thereby enhancing ORR performance. When applied in ZABs, Fe–N_5_ SACs delivered a high open-circuit voltage (OCV), an excellent peak power density, and stable long-term continuous discharge. This wood-derived spatial and Fe^3+^ coordination dual-confinement strategy is effective in creating a hierarchical porous architecture with in situ heteroatom doping to improve the electrocatalytic activity and reaction kinetics of SACs.

## Results and Discussion

### Synthesis and characterization of Fe–N_5_

**Fig. 1.**
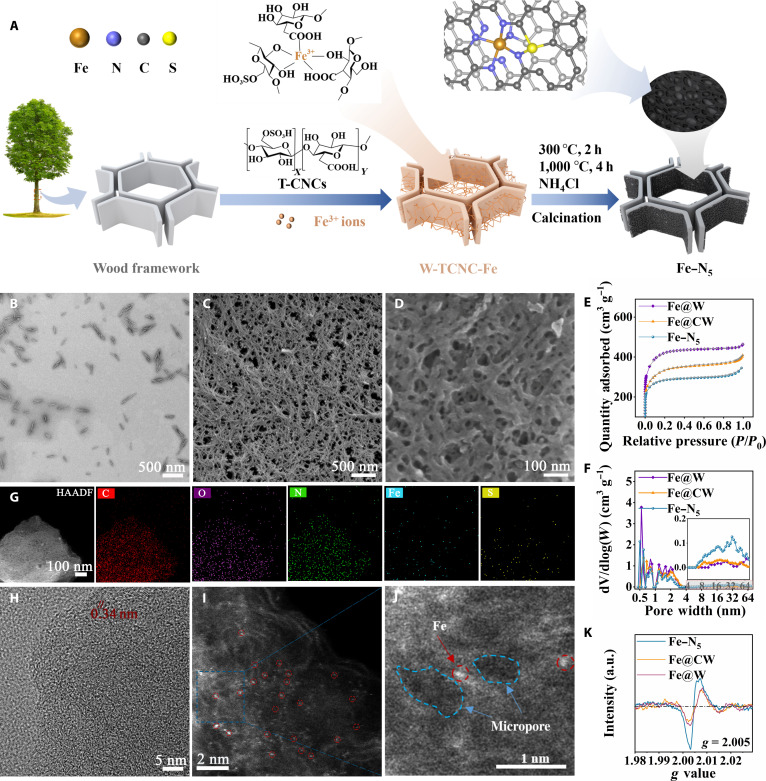
Synthesis and structural characterization. (A) Synthetic schematic of Fe–N_5_. (B) Transmission electron microscopy (TEM) image of 2,2,6,6-tetramethylpiperidin-1-oxyl (TEMPO)-modified cellulose nanocrystals (CNCs) (T-CNCs). Scanning electron microscopy (SEM) images of (C) W-TCNC-Fe and (D) Fe–N_5_. (E) N_2_ sorption isotherms and (F) pore size distributions of Fe–N_5_, Fe@CW, and Fe@W (inset: magnification of the boxed region). (G) High-angle annular dark-field scanning transmission electron microscopy (HAADF-STEM) image and energy-dispersive x-ray spectroscopy (EDX) elemental mapping of Fe–N_5_. (H) High-resolution TEM image of Fe–N_5_. (I) Aberration-corrected (AC) HAADF-STEM of Fe–N_5_. (J) Magnified AC HAADF-STEM of Fe–N_5_. (K) Electron paramagnetic resonance (EPR) of Fe–N_5_, Fe@CW, and Fe@W.

An Fe–N–C catalyst with hierarchical hollow-interconnected porous architectures was fabricated via the self-assembly of TEMPO-modified carboxylated CNCs (T-CNCs) under the dual-confinement effect of wood framework and Fe^3+^ ions, followed by a calcination process, as illustrated in Fig. 1A. To enhance the coordination ability of CNCs with Fe^3+^ ions, a 2-step functionalization strategy was implemented to introduce –COOH groups onto the sulfuric acid-hydrolyzed CNCs (S-CNCs) [20]. This process involved hydrochloric acid-mediated surface activation at 80 °C, followed by TEMPO-mediated oxidation, as depicted in Fig. S1a. The x-ray diffraction (XRD) patterns of S-CNCs and T-CNCs (Fig. S1b and c) exhibit 3 peaks at 14.8°, 16.1°, and 22.8°, which correspond to the $\left(1\bar{1}0\right)$, (110), and (020) crystallographic planes [21], respectively. Compared with that in S-CNCs, the reduced intensity of the diffraction peak at 16.1° in T-CNCs indicates that the crystalline structure of S-CNCs is perturbed by carboxylation treatment [20]. Fourier transform infrared spectroscopy (Fig. S1d) provides further evidence for the successful incorporation of –COOH. Relative to S-CNCs, T-CNCs show a diminished intensity of the –OH peak at 3,328 cm^−1^, which arises from the replacement of partial –OH groups with –COOH groups. Concomitantly, a new peak emerges at 1,731 cm^−1^, corresponding to the –C=O stretching vibration of –COOH, providing direct evidence for the oxidation of –OH groups. Notably, both T-CNCs and S-CNCs exhibit a peak at 812 cm^−1^, corresponding to the C–O–S stretching vibration of –OSO_3_H groups [22]. This result confirms that the –OSO_3_H groups remain intact on the T-CNC surface after the 2-step functionalization, providing a S source for subsequent in situ S doping. Transmission electron microscopy (TEM) images (Fig. 1B and Fig. S2) show that T-CNCs and S-CNCs both maintain good dispersion in aqueous solution, presenting as rodlike nanostructures with uniform dimensions. The well-dispersed nature of CNCs is critical for their subsequent self-assembly, as it ensures sufficient interaction with surrounding coordination sites (e.g., Fe^3+^ ions and wood cell wall functional groups) to form a homogeneous and stable network structure. Subsequently, the wood framework impregnated with T-CNCs was transferred to an ethanol solution containing Fe^3+^ ions. As Fe^3+^ ions diffuse through the natural hierarchical channels of the wood, the dual-confinement-driven self-assembly of T-CNCs is initiated, and the wood framework provides spatial confinement to guide the macroscopic arrangement of T-CNCs, while Fe^3+^ ions act as coordination cross-linkers to mediate the microscopic assembly of T-CNCs via electrostatic interactions and coordinate bonds (e.g., C–O···Fe^3+^). To eliminate residual unanchored Fe^3+^ ions and avoid structural damage caused by ice crystal growth during freeze-drying, the ethanol within the wood framework was replaced with a *tert*-butanol aqueous solution [23], yielding the intermediate composite W-TCNC-Fe. The W-TCNC-Fe composite was first subjected to low-temperature carbonization at 300 °C. This step serves 2 key purposes, which are stabilizing the layered porous network formed by T-CNCs within the wood pores by promoting the partial carbonization of cellulose and the consolidation of hydrogen bonds between T-CNCs and wood cell walls, and facilitating the homogeneous mixing of the carbonaceous matrix with NH_4_Cl (a nitrogen source) during subsequent cogrinding to lay the foundation for uniform N doping. Finally, high-temperature carbonization at 1,000 °C was performed to fabricate the Fe–N_5_ catalyst. During this process, NH_4_Cl decomposes to release NH_3_ and HCl gases. NH_3_ acts as a nitrogen source to dope N into the carbon network (forming pyridinic N, pyrrolic N, and graphitic N), while HCl etches the carbon frameworks to generate abundant micropores. These micropores function as anchoring sites for Fe atoms, effectively suppressing Fe agglomeration and enabling the generation of atomically dispersed Fe sites [24]. To decouple the contributions of each component (wood framework, CNCs, Fe^3+^ ions, and N source) to the hierarchical porous structure and the microenvironment of single-atom Fe, a series of control samples were prepared, which were N@TCW, Fe@TCW, Fe@CW, Fe@W, Fe@H80, Fe@H100, and Fe–N(100). Specifically, N@TCW was synthesized without adding Fe^3+^ ions (i.e., no Fe source). Fe@TCW was synthesized without adding NH_4_Cl (i.e., no N source). Fe@CW was synthesized using S-CNCs instead of T-CNCs. Fe@W was synthesized by directly introducing Fe^3+^ ions and NH_4_Cl into the wood framework (without CNCs). Fe@H80 and Fe@H100 were synthesized by replacing T-CNCs with CNC-H80 and CNC-H100, respectively. Fe–N(100) was synthesized using CNC-100 instead of T-CNCs.

Figure S3 shows the cross-sectional architecture of delignified balsa wood. Its inherent hierarchical honeycomb-like pores act as a 3D spatial template to create a confined microenvironment that guides the self-assembly of T-CNCs. To clarify the role of dual confinement from the wood framework and Fe^3+^ ions in structure formation, control experiments on T-CNCs’ self-assembly were conducted under different conditions. Without both the wood framework and Fe^3+^ ions, T-CNCs self-assemble into a dense layered structure with no observable pores (Fig. S4). This outcome stems from random stacking driven by unregulated intermolecular hydrogen bonding. With only the wood framework and no Fe^3+^ ions, T-CNCs form a loose fibrous network (Fig. S5) but lack Fe^3+^-mediated cross-linking, which leads to poor structural integrity and irregular pore distribution. With only Fe^3+^ ions and no wood framework, T-CNCs aggregate into a compact structure without large-scale pores (Fig. S6). This is because Fe^3+^ coordination alone causes excessive cross-linking without spatial guidance. These results confirm the indispensable role of the wood framework in directing T-CNCs’ self-assembly into a porous network. Specifically, under the dual-confinement effect, the wood framework provides spatial guidance and Fe^3+^ ions act as coordination cross-linkers, enabling T-CNCs to assemble into a uniformly distributed and ordered porous architecture (Fig. 1C). Abundant hydrogen bonds also form between the –OH groups on wood cell walls and T-CNCs’ functional groups including –OH, –COOH, and –OSO_3_H. These bonds reinforce the structural stability of the T-CNC network and preserve its porous structure during subsequent high-temperature carbonization.

After carbonization at 1,000 °C, the Fe–N_5_ catalyst retains the hierarchical 3D interconnected porous architecture (Fig. 1D), a key feature for ORR performance. In contrast, Fe@W, which is prepared by directly loading Fe^3+^ ions onto wood without T-CNCs, has no observable porous structure (Fig. S7). This confirms the necessity of T-CNCs for constructing the porous carbon matrix. Notably, Fe@TCW synthesized without the N precursor NH_4_Cl still forms abundant pores (Fig. S8a). These pores are derived from the preformed porous architecture of W-TCNC-Fe, which is stabilized by the dual-confinement effect of the wood framework and Fe^3+^. This indicates that the porous structure is primarily determined by the dual-confinement effect of the wood framework and Fe^3+^ rather than N doping. In stark contrast, N@TCW prepared without Fe^3+^ ions shows no discernible porosity (Fig. S8b). Without Fe^3+^-mediated cross-linking, T-CNCs fail to form a stable porous network within the wood framework and collapse during carbonization. Fe@CW synthesized using S-CNCs instead of T-CNCs displays a stacked and collapsed porous network (Fig. S9). This phenomenon arises from the weaker coordination ability of –OSO_3_H groups on S-CNCs toward Fe^3+^ ions compared to –COOH groups on T-CNCs. Inductively coupled plasma mass spectrometry (ICP-MS) results (Table S1) support this observation. The Fe content of Fe–N_5_ is 0.61%, approximately twice that of Fe@CW, which is 0.33%. This confirms that –COOH groups enable stronger Fe^3+^ ion coordination and higher Fe loading. Furthermore, pore structure of catalysts is then revealed by N_2_ adsorption–desorption isotherms and pore size distribution. As shown in Fig. 1E, all catalysts, including Fe–N_5_, Fe@CW, and Fe@W, show sharp increases in the low-relative-pressure region, indicating the existence of microporous structures that favor the anchoring of Fe single atoms. Different from Fe@W, both Fe–N_5_ and Fe@CW exhibit representative type IV isotherms with H_4_ hysteresis loops in the high-relative-pressure range, demonstrating the simultaneous presence of mesoporous channels. The pore size distribution provided by the Brunauer–Emmett–Teller and mercury intrusion porosimetry methods (Fig. 1F and Fig. S10) further demonstrates that Fe–N_5_ possesses a hierarchical pore structure with micropores (0.46 to 3.00 nm), mesopores (5.00 to 80.00 nm), and macropores (121.00 nm). Consequently, this distinctive Fe–N_5_ catalyst featuring micropores and mesopores that densely penetrate the carbon matrix rich in macropores is constructed, which could not only maximize the exposure of actives sites but also ensure efficient mass transport and electron transfer at the 3D interfaces [24].

The XRD patterns of Fe–N_5_, Fe@TCW, Fe@CW, and Fe@W exhibit 2 broad peaks at 25° and 44° (Fig. S11), corresponding to the (002) and (101) crystal planes of graphitized carbon, respectively. No metallic Fe or Fe compounds in Fe–N_5_, Fe@CW, and Fe@W are observed. In contrast, Fe@TCW displays a distinct metallic Fe peak near 44°, demonstrating that the atomic-level Fe dispersion requires a coordination structure formed with N atoms that are derived from NH_4_Cl. High-resolution TEM (Fig. 1H) further confirms no Fe nanoparticles in Fe–N_5_. The lattice fringe is estimated to be 0.34 nm, which corresponds to the (002) planes of graphitic carbon, in accordance with XRD results. As shown in Fig. S12, no obvious iron nanoparticles are observed in Fe@CW and Fe@W.

High-angle annular dark-field scanning transmission electron microscopy (HAADF-STEM) and corresponding energy-dispersive x-ray spectroscopy (EDX) elemental mapping (Fig. 1G and Figs. S13 and S14) also indicate that Fe atoms in Fe–N_5_, Fe@CW, and Fe@W are uniformly dispersed within the N, S-codoped carbon matrix without agglomeration. The aberration-corrected high-angle annular dark-field scanning transmission electron microscopy (AC HAADF-STEM) images (Fig. 1I) provide direct visualization of atomically dispersed Fe as bright spots. The local magnified view (Fig. 1J) shows that Fe atoms are localized at the edges of micropores within the carbon matrix, which might modulate the charge density distribution and electronic structures of active centers, thus optimizing intermediates’ adsorption configuration [25]. The disordered structure is observed by Raman spectroscopy (Fig. S15). The D band at 1,350 cm^−1^ is attributed to sp^3^-hybridized defective carbon, and the G band at 1,580 cm^−1^ is attributed to sp^2^-hybridized graphitic carbon. Fe–N_5_ exhibits an *I*_D_/*I*_G_ ratio of 1.04, which is similar to that of Fe@TCW (1.05) but significantly higher than that of N@TCW (1.01). This higher ratio indicates a greater density of carbon defects in the T-CNC-Fe^3+^ coordination network. Electron paramagnetic resonance spectra (Fig. 1K) show a characteristic signal at *g* = 2.005 for Fe–N_5_, Fe@CW, and Fe@W, indicating structural defects in all catalysts. Notably, Fe–N_5_ exhibits a much higher signal intensity than Fe@CW and Fe@W, confirming more defect sites [26].

The chemical composition and oxidation states of Fe–N_5_ and related samples were investigated by high-resolution x-ray photoelectron spectroscopy (XPS). The high-resolution N 1s spectra (Fig. S16a) of Fe–N_5_, Fe@CW, and Fe@W reveal 5 peaks centered at 398.28, 399.26, 400.50, 401.56, and 403.40 eV, which are attributed to Fe–N, pyridinic N, pyrrolic N, graphitic N, and oxidized N, respectively. As shown in Fig. S16b, oxidized N remains relatively constant across the 3 samples. Compared with Fe@CW and Fe@W, Fe–N_5_ exhibits increased graphitic N, of which graphitic N facilitates electron transfer within the carbon framework. Compared to Fe@W, both Fe–N_5_ and Fe@CW exhibit increased Fe–N content and reduced proportions of pyridinic N and pyrrolic N. This arises from the increased amount of Fe due to the coordination of functional groups on CNCs with Fe^3+^ ions, which requires more pyridinic N and pyrrolic N to generate isolated Fe–N*_x_* sites. Moreover, the carboxylation of S-CNCs further facilitates the involvement of both pyridinic N and pyrrolic N in the coordination with Fe atoms. The S 2p spectrum (Fig. S17) of Fe–N_5_ exhibits 2 distinct peaks at 164.29 and 165.54 eV, assigned to thiophene-like C–S–C species (2p_3/2_ and 2p_1/2_), along with weak peaks at 168.53 and 169.59 eV, corresponding to oxidized –SO*_x_*^−^ species. Compared with Fe@CW, no signal of Fe–S bonds is detected in Fe–N_5_, confirming the absence of Fe–S coordination. Furthermore, both the high-resolution N 1s and S 2p XPS spectra of the Fe–N_5_ moiety exhibit additional characteristic peaks, which are assigned to N–S bonding species. This spectral feature provides direct evidence that the S atom is spatially adjacent to the N atom in the carbon matrix, an arrangement that originates from the in situ doping of S (derived from the residual –OSO_3_H groups of T-CNCs) and its subsequent interaction with N-coordinated Fe sites during high-temperature carbonization. The characteristic peaks in these spectra correspond to S species bonded to carbon, further validating the in situ sulfur doping process derived from residual –OSO_3_H groups on T-CNCs. Meanwhile, O 1s XPS further confirms the absence of Fe–O bonding in Fe–N_5_ (Fig. S18). Fe 2p XPS spectra reveal that the 2p_3/2_ peaks of Fe–N_5_, Fe@CW, and Fe@W are located at 709.29 eV (Fe^2+^) and 712.10 eV (Fe^3+^), 710.36 eV (Fe^2+^) and 713.40 eV (Fe^3+^), and 709.68 eV (Fe^2+^) and 713.09 eV (Fe^3+^), respectively, accompanied by satellite peaks—these features indicate the partially oxidized state of Fe (Fig. S19). Compared with Fe@CW, no characteristic peak corresponding to Fe–S species is detected in Fe–N_5_, demonstrating that TEMPO-mediated carboxylation effectively suppresses the formation of Fe–S coordination structures. Notably, the Fe 2p_3/2_ peak of Fe–N_5_ exhibits the lowest binding energy compared to those of Fe@CW and Fe@W. This observation suggests that the coordination structure of Fe–N_5_ facilitates greater electron accumulation around the Fe centers, which favors oxygen intermediate uptake and release throughout the ORR process, thus optimizing catalytic kinetics. Furthermore, peaks indicative of metallic Fe-based species are absent in Fe–N_5_, evidencing the successful formation of atomically dispersed Fe sites within the carbon matrix.

### ORR performance

**Fig. 2.**
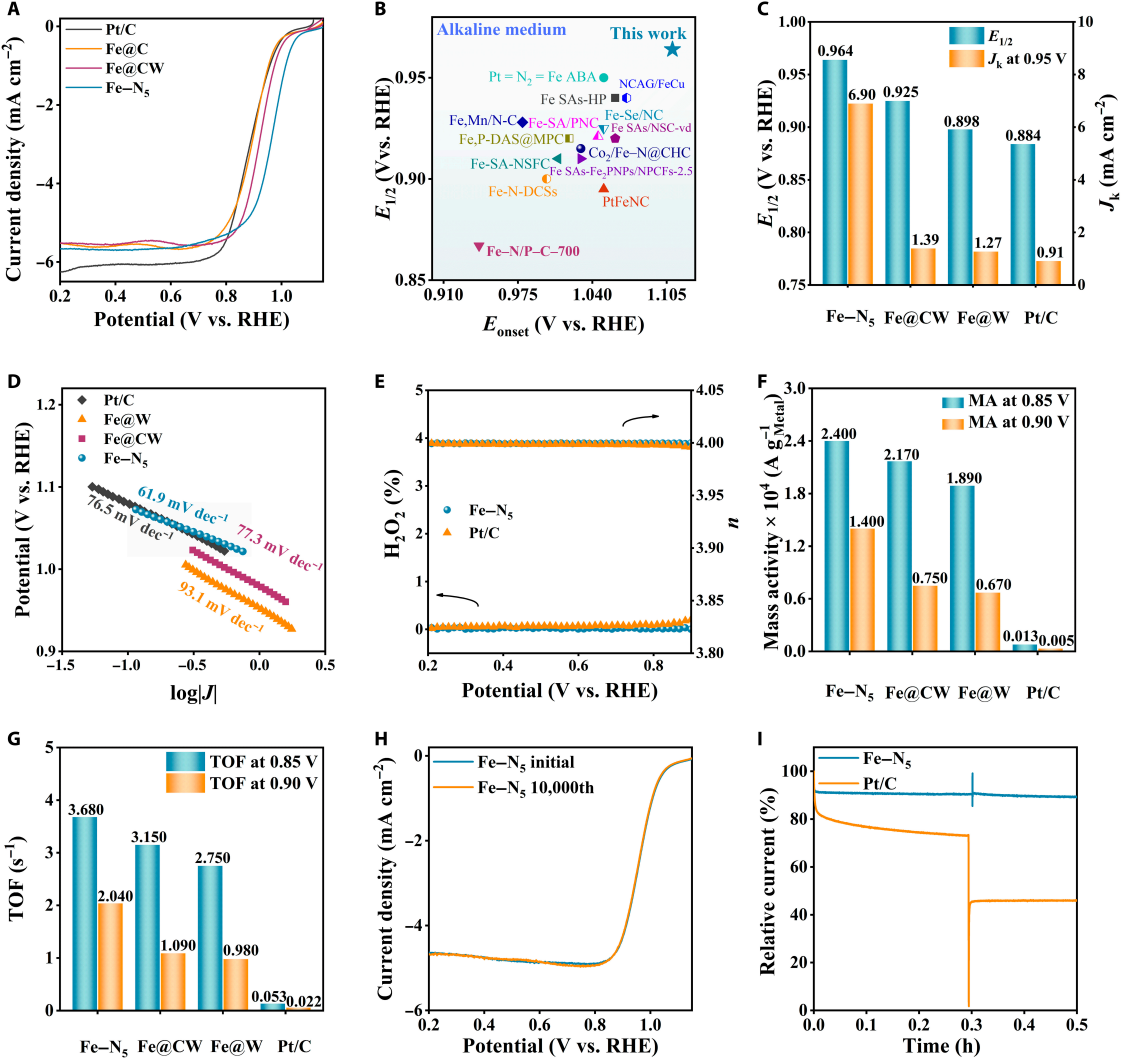
Electrochemical oxygen reduction reaction (ORR) performance. (A) linear sweep voltammetry (LSV) curves. (B) Benchmarking the alkaline ORR activity of Fe–N_5_ against catalysts reported in the literature. (C) Comparison of *E*_1/2_ and *J*_k_ at 0.95 V. (D) Tafel plots for different catalysts. (E) H_2_O_2_ selectivity and *n* of Fe–N_5_ and Pt/C. (F) Mass activity and (G) turnover frequency (TOF) values of Fe–N_5_ and related samples at 0.85 and 0.90 V. (H) Durability test of Fe–N_5_ for 10,000 cycles. (I) Methanol resistance *i*–*t* test.

The ORR activities of all samples were evaluated in an O_2_-saturated 0.1 M KOH electrolyte. First, the cyclic voltammetry (CV) curves of the catalysts (Fig. S20) exhibit distinct oxygen reduction peaks. Among these, Fe–N_5_ shows the most positive cathodic peak potential at 0.938 V, a key indicator of its superior ORR performance relative to those of the other catalysts. The linear sweep voltammetry curves (Fig. 2A and Fig. S21a) further confirm the outstanding ORR activity of Fe–N_5_, displaying a half-wave potential (*E*_1/2_) of 0.964 V and an onset potential (*E*_onset_) of 1.110 V, which surpass those of commercial Pt/C (*E*_1/2_ of 0.884 V and *E*_onset_ of 1.040 V), Fe@CW (0.925 and 1.080 V), Fe@W (0.898 and 1.080 V), and Fe–N(100) (0.940 and 1.080 V). Notably, the superior performance of Fe–N_5_ over Fe–N(100) validates the positive contribution of in situ S doping toward ORR activity. Fe–N(100) is derived from the confined self-assembly of CNC-100 without –SO_3_H moieties (Fig. S21b). Although CNC-100 contains abundant –COOH groups and achieves a higher Fe loading of 0.85% (Table S1), the lack of S doping renders its catalytic activity inferior to that of Fe–N_5_. In addition, Fe@H80 exhibits a lower Fe loading of 0.45% than that of Fe@H100 (0.51%) (Table S1), while both catalysts deliver a comparable *E*_1/2_ of 0.95 V (Fig. S21d). This phenomenon mainly originates from the much higher S content in Fe@H80 (0.85%) than that in Fe@H100 (0.02%), further indicating that S doping effectively enhances ORR activity by modulating the electronic structure of active sites. Furthermore, Fe–N(100) exhibits a markedly higher activity than Fe@W, highlighting the indispensable role of the hierarchical porous structure (Fig. S21c) fabricated via the dual-confinement strategy in mass transport and exposure of active sites. Notably, a comprehensive comparison with previously reported M–N–C catalysts (Fig. 2B and Table S9) reveals that the ORR performance of Fe–N_5_ stands among the top tier of non-precious-metal catalysts. The kinetic current density (*J*_k_) (Fig. 2C) of Fe–N_5_ at 0.95 V reaches 6.90 mA cm^−2^, which is 7.6 times that of Pt/C (0.91 mA cm^−2^) and significantly exceeds those of Fe@CW (1.39 mA cm^−2^) and Fe@W (1.27 mA cm^−2^). This demonstrates its outstanding catalytic performance and the superiority of the dual-confinement effect for establishing Fe–N–C active sites. The fast ORR kinetics of Fe–N_5_ is further evidenced by its Tafel slope of 61.9 mV dec^−1^ as shown in Fig. 2D, which is lower than those of Fe@CW (77.3 mV dec^−1^), Fe@W (93.1 mV dec^−1^), and Pt/C (76.5 mV dec^−1^). Moreover, Fig. S22 illustrates that Fe–N_5_ possesses the smallest electrochemical double-layer capacitance (*C*_dl_) of 17.91 mF cm^−2^, while Fe@CW shows the highest *C*_dl_ of 23.93 mF cm^−2^, followed by Fe@W (19.32 mF cm^−2^). This result confirms that the electrochemical active surface area (ECSA) plays a nonprimary role in driving the improved ORR performance of Fe–N_5_, pointing instead to the superior intrinsic activity of its Fe–N active sites.

Rotating ring-disk electrode (RRDE) measurements (Fig. 2E) demonstrate that the electron transfer number (*n*) of Fe–N_5_ is 3.99 to 4.00, accompanied by an exceptionally low H_2_O_2_ yield of less than 0.5%, unambiguously demonstrating the high selectivity for the 4*e*^−^ ORR process. This is further confirmed by the Koutecký-Levich (K-L) plots in Fig. S23, of which *n* is calculated to be 4.02 over the potential range of 0.3 to 0.7 V. To evaluate the intrinsic activity of the catalysts, the mass activity (MA) and turnover frequency (TOF) were calculated. The MA values (Fig. 2F and Table S2) of Fe–N_5_ at 0.85 and 0.90 V are 2.40 × 10^4^ and 1.40 × 10^4^ A g_Fe_^−1^, respectively, which are 184 and 259 times as much as those of Pt/C (130.00 and 54.00 A g_Pt_^−1^) and exceed those of Fe@CW (2.17 × 10^4^ and 7.50 × 10^3^ A g_Fe_^−1^) and Fe@W (1.89 × 10^4^ and 6.70 × 10^3^ A g_Fe_^−1^). The TOF values (Fig. 2G and Table S3) of Fe–N_5_ at 0.85 and 0.90 V are 3.680 and 2.040 s^−1^, respectively, corroborating the higher activity than those of Pt/C (0.053 and 0.022 s^−1^), Fe@CW (3.150 and 1.090 s^−1^), and Fe@W (2.750 and 0.980 s^−1^). Electrochemical nitrite stripping (Fig. S24 and Table S4) is employed to determine the active site density and more precise TOF values [27]. The site density values for Fe–N_5_, Fe@CW, and Fe@W are determined to be 1.42 × 10^19^, 7.84 × 10^18^, and 1.12 × 10^19^ sites g^−1^, respectively. The TOF value of Fe–N_5_ is estimated to be 3.04 *e*^−^ site^−1^ s^−1^, higher than those of Fe@CW (1.11 *e*^−^ site^−1^ s^−1^) and Fe@W (0.71 *e*^−^ site^−1^ s^−1^). These results collectively confirm that the unique Fe–N moiety, constructed via the dual-confinement effect of T-CNCs, is responsible for the enhanced ORR efficiency.

The stability of the electrocatalysts is assessed by continuous 10,000 CV cycles across a potential window of 0.165 to 1.165 V. After 10,000 CV cycles, Fe–N_5_ exhibits negligible decay in both *E*_1/2_ and limiting current density (Fig. 2H). In contrast, commercial Pt/C displays a 24-mV decay in *E*_1/2_ after only 5,000 CV cycles (Fig. S25). Chronoamperometric (*i*–*t*) tests (400 rpm, O_2_-saturated 0.1 M KOH) (Fig. S26) further validate the excellent durability of Fe–N_5_, which retains 92.3% of its initial current after 10 h, superior to that of Pt/C (78.7%). The outstanding catalytic activity and stability of Fe–N_5_ suggest that both pyridinic N, which contributes to superior stability, and pyrrolic N, which is responsible for enhanced catalytic activity, participate in the coordination of the central Fe atom [28]. Additionally, methanol tolerance tests (Fig. 2I) demonstrate the remarkable methanol tolerance of Fe–N_5_, demonstrating the potential application for direct methanol fuel cells.

### Identifying the coordination structure of Fe active sites

**Fig. 3.**
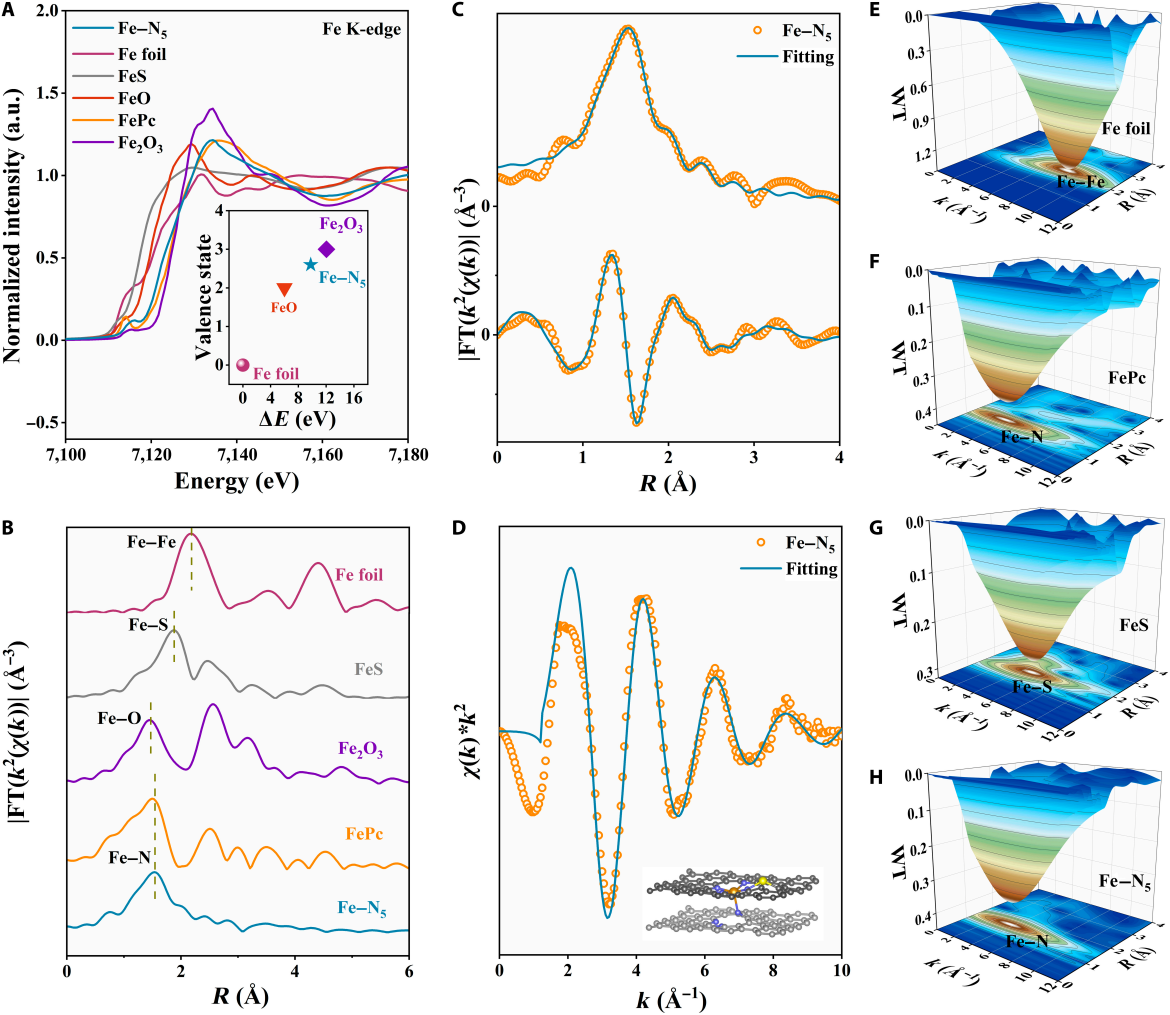
X-ray absorption spectroscopy (XAS) analysis of Fe–N_5_ and as-prepared catalysts. (A) Fe K-edge x-ray absorption near-edge structure (XANES) spectra of Fe–N_5_ (the inset shows the fitted average oxidation states of Fe from XANES spectra). (B) *k*^2^-weighted Fourier transform (FT) spectra of Fe at *R* spaces. Fe K-edge extended x-ray absorption fine structure (EXAFS; points) and the curve fitting (line) for Fe–N_5_ shown in (C) *R* space and (D) *k*^2^-weighted *k* space. (E to H) Wavelet transform EXAFS (WT-EXAFS) of Fe K-edge for Fe foil, FePc, FeS, and Fe–N_5_, respectively.

To decode the chemical state and local coordination environment of the central Fe site at the atomic scale, synchrotron-based XAS measurements were conducted. Figure 3A presents the Fe K-edge x-ray absorption near-edge structure (XANES) spectrum of Fe–N_5_ alongside those of reference samples including Fe foil, FeS, FePc, FeO, and Fe_2_O_3_. FePc is a model compound with square-planar Fe–N_4_ coordination. A characteristic pre-edge peak is observed at 7,114 eV for FePc, and this peak corresponds to the 1s → 4pz electronic transition. It serves as a spectral fingerprint sensitive to the local symmetry of the central Fe atom [29], providing a benchmark for comparing Fe coordination environments. For Fe–N_5_, the pre-edge peak (7,116 eV) exhibits a slight rightward shift and a reduced intensity, indicating the presence of a distorted Fe–N configuration [30]. This distortion may originate from adjustments to the Fe coordination number, heteroatom doping such as S doping, or defects in the carbon matrix [31]. The absorption edge energy of Fe–N_5_ is intermediate between those of FeO (Fe^2+^) and Fe_2_O_3_ (Fe^3+^). It is also closer to that of FePc, suggesting an intermediate Fe oxidation state between +2 and +3. Compared with that in FePc, the Fe species in the Fe–N_5_ catalyst exhibits a lower oxidation state. This reduction in Fe oxidation state results from the enhanced electron-donating effect of the N, S-codoped carbon matrix, where the introduction of in situ S doping modulates the electron delocalization within the carbon framework. Specifically, S atoms, with a lower electronegativity than N, can alter the charge distribution at Fe–N_5_ active sites, promoting electron transfer away from the carbon matrix toward the central Fe atoms and thus lowering the Fe oxidation state [32]. This electron-enriched Fe environment is critical for optimizing the adsorption strength of oxygen-containing intermediates during the ORR, ultimately boosting catalytic kinetics. Quantitative fitting of Fe K-edge XANES by comparing with standard samples (FeO and Fe_2_O_3_) reveals an average oxidation state of approximately +2.6 for Fe–N_5_ (inset of Fig. 3A), which coincides with XPS results (Fig. S19). The *k*^2^-weighted Fourier transform spectra from extended x-ray absorption fine structure (EXAFS) spectroscopy (Fig. 3B) further clarify a dominant peak of Fe–N_5_ at 1.53 Å, slightly higher than that of FePc, which originates from the first-shell Fe–N scattering path. This observation suggests the possible presence of axial N coordination in Fe–N_5_, which elongates the Fe–N bond length within the Fe–N_5_ plane. The absence of Fe–S (1.87 Å) and Fe–Fe (2.20 Å) peaks can be observed, confirming the atomic dispersion of Fe, consistent with XRD (Fig. S11a) and HAADF-STEM (Fig. 1I) results. Quantitative fitting of the EXAFS spectra in *R* and *k* spaces gives an *R*-factor of only 0.010 with excellent fitting quality and indicates a first-shell Fe–N coordination number of 5.30 ± 0.2 with an average Fe–N distance of 2.03 Å, suggesting Fe–N_5_ coordination (Fig. S27 and Table S5), which is distinctly different from the 4-coordinate Fe–N bond length (1.94 Å) in FePc. Combined with the pre-edge XANES features, the axial pyrrolic N coordinated to 4 pyridinic N in FeN_5_ moieties is inferred, which coincides with the well-fitting results of K-edge EXAFS with the proposed model in both *R* and *k* spaces (Fig. 3C and D). The wavelet transform contour maps of Fe–N_5_ reveal a peak at 3.60 Å^−1^ (Fig. 3H), assigned to the Fe–N scattering path. While Fe foil exhibits a maximum intensity at 7.60 Å^−1^ (Fig. 3E), aligning with the Fe–Fe bond, Fe_2_O_3_, FePc, and FeS show maxima at 3.30 Å^−1^ (Fe–O) (Fig. S28), 3.40 Å^−1^ (Fe–N) (Fig. 3F), and 4.75 Å^−1^ (Fe–S) (Fig. 3G), respectively. These results conclusively corroborate that the atomically dispersed iron sites reside in a unique Fe–N_5_ coordination configuration.

### DFT calculations

**Fig. 4.**
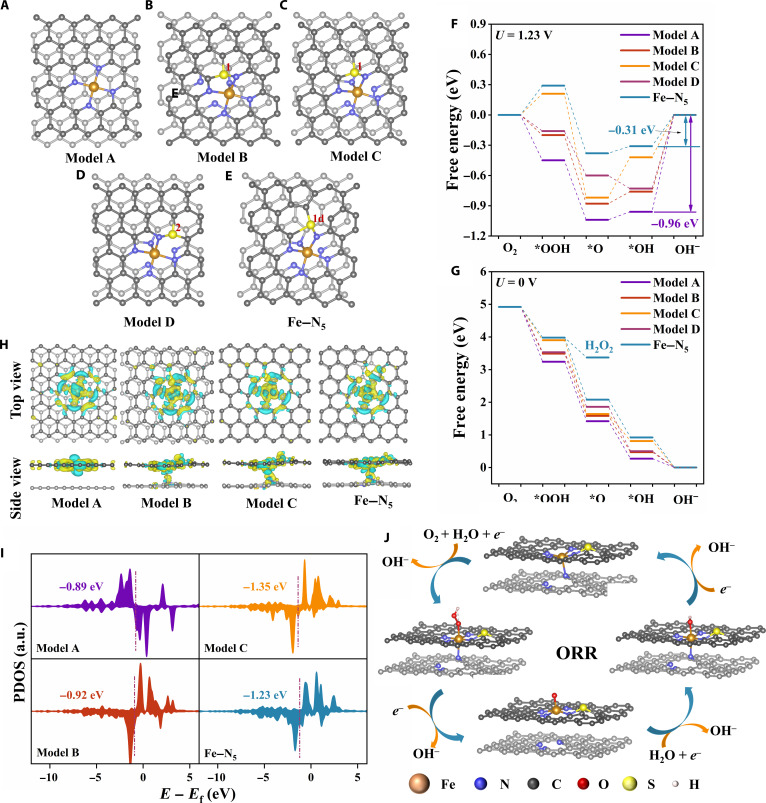
Density functional theory (DFT) theoretical calculations. (A to E) Illustration of model A, model B, model C, model D, and Fe–N_5_. Dark gray: first carbon layer. Light gray: second carbon layer. Gold: Fe. Blue: N. Yellow: S. Free energy diagram of model A, model B, model C, model D, and Fe–N_5_ at (F) *U* = 1.23 V and (G) *U* = 0 V. (H) Differential charge density diagram of model A, model B, model C, and Fe–N_5_ in top and side views (yellow and cyan represent the increase and decrease in electronic density, respectively). (I) Fe 3d partial density of states (PDOS) of model A, model B, model C, and Fe–N_5_. (J) A schematic of the proposed oxygen reduction reaction (ORR) pathway for Fe–N_5_.

To elucidate the atomic-level mechanism underlying the superior activity of the Fe–N_5_ catalyst, density functional theory (DFT) calculations were conducted. Multiscale characterizations including scanning electron microscopy, HAADF imaging, Raman spectroscopy, XPS, and XAS reveal that Fe–N_5_ possesses a hierarchically porous structure, with Fe atomic centers situated in a 5-coordination environment at the micropore edges, along with the presence of S doping. To systematically investigate the effect of structural geometry on the electronic properties and catalytic behavior of Fe active sites, 4 distinct configurations of Fe-coordinated moieties were constructed on the first carbon layer (denoted as layers 1a to 1d), with the classic 4-fold pyridinic N-coordinated Fe moiety (Fe–N_4_) embedded in a pristine carbon network (layer 1a) serving as the reference system (Fig. S29). Layer 1b features 4 pyridinic N atoms coordinated to a central Fe atom, with 1 S atom incorporated into the same 5-membered ring as the Fe center (Fig. S29). For layer 1c, the S atom is positioned within the 6-membered ring that contains the central Fe atom (Fig. S29), differing from layer 1b in the cyclic environment of the doped S. Layer 1d is derived from layer 1b by introducing an adjacent micropore into the first carbon network, with the pore localized near the S atom (hereafter referred to as the S1 site) to mimic the hierarchical porous feature of the catalyst (Fig. S29). Correspondingly, 3 configurations are designed for the second carbon layer (light gray, Fig. S29) to explore the influence of axial coordination on Fe sites. Layer 2e shows a pristine carbon network without additional heteroatoms, used to replicate the nonaxial coordination environment (Fig. S29). Layer 2f exhibits a carbon network with an axial pyridinic N atom that coordinates to the central Fe, enabling the formation of an Fe–N_5_ configuration (Fig. S29). Meanwhile, layer 2g is similar to layer 2f, but with an axial pyrrolic N atom instead of pyridinic N, to compare the effect of axial N species on Fe coordination (Fig. S29). These first and second carbon layer configurations are further combined to construct 5 representative models for subsequent analysis. For model A, it represents the traditional Fe–N_4_ coordination structure, which is composed of layer 1a and layer 2e (Fig. 4A). Model B combines layer 1b and layer 2f, as presented in Fig. 4B. For model C, it is formed by layer 1b and layer 2g (Fig. 4C). Model D consists of layer 1c and layer 2g, as shown in Fig. 4D. Model E is composed of layer 1a and layer 2g, as shown in Fig. S30a. For the target configuration, that is, the Fe–N_5_ model, it is constructed by layer 1d and layer 2g (Fig. 4E).

Figure 4F demonstrates a strong correlation between Fe site geometry and catalytic behavior. The 4*e*^−^ ORR process primarily involves the adsorption of 3 key intermediates: *OOH, *O, and *OH. At *U* = 1.23 V (Fig. 4F, Fig. S30b, and Table S6), the last step of ORR (*OH desorption) is the most endothermic on all catalyst models and thus is the reaction-determining step (RDS). The Fe–N_5_ model shows the lowest energy barrier of 0.31 eV among the catalysts, that is, model A (0.96 eV), model B (0.76 eV), model C (0.42 eV), model D (0.73 eV), and model E (0.89 eV). In contrast to model C, Fe–N_5_ shows a reduced energy barrier, confirming that micropores optimize intermediate adsorption via electronic structure regulation. Compared with model A, model E possesses a lower energy barrier, indicating that axial pyrrolic N coordination regulates the electronic structure of Fe sites and weakens oxygen-containing intermediate adsorption. Model C delivers a far lower barrier than model E, revealing strong electronic synergy of S doping with the FeN_5_ framework to tune the active site electronic structure and boost intrinsic catalytic activity. As indicated in Fig. 4G, at *U* = 0 V, all ORR steps from O_2_ to OH^−^ are spontaneous exothermic on Fe–N_5_. The significant difference between the free energy for *OOH → *O (1.9 eV) and the H_2_O_2_ formation pathway (0.61 eV) further confirms the preferential selection of the 4*e*^−^ pathway on Fe–N_5_ (Fig. 4J) and effective suppression of by-product generation (Table S7). Bond length analysis (Figs. S31, S32, and S34 and Table S8) provides insight into the stability of Fe–N coordination. The Fe–N_5_ bond length in model B with axial pyridinic N coordination is measured as 2.40 Å, significantly longer than that in pyrrolic N-coordinated Fe (model C, 2.16 Å, and Fe–N_5_, 2.12 Å), indicating the superior stability of the Fe–N_5_ bond in the catalysts with axial pyrrolic N coordination.

As revealed by the differential charge density plot (Fig. 4H), it shows large nonuniform charge distribution at the Fe sites on Fe–N_5_. Compared to model A, S doping in Fe–N_5_ is beneficial for adsorbing electrons [31] and donating electrons to Fe, resulting in a lower oxidation state of Fe, which is consistent with the XAS and XPS results. Meanwhile, compared to model C, the existence of a micropore near the S_1_ site slightly decreases the charge density at the Fe sites, which may be due to the accelerated shutting of electrons by micropores [24]. The d-band center theory is employed to analyze the binding affinity of Fe sites for oxygen intermediates as shown in Fig. 4I. Compared to model A (−0.89 eV), the d-band centers of Fe–N_5_, model B, and model C shift to −1.23, −0.92, and −1.35 eV, respectively. A downshift in the d-band center weakens the binding energy between the Fe site and *OH, thereby promoting *OH desorption. Notably, the introduction of a micropore near the S_1_ site when comparing Fe–N_5_ with model C induces a slight rightward shift of the d-band center. This shift balances the adsorption/desorption strength of oxygen intermediates at the Fe site. It avoids overly strong *OH adsorption, which would hinder desorption or overly weak adsorption, which would impair intermediate activation. Collectively, these DFT results demonstrate that the unique configuration of Fe–N_5_ contributes to its excellent ORR performance. This configuration consists of an Fe–N_5_ moiety with axial pyrrolic N coordinated to 4 equatorial pyridinic N, a S atom in the adjacent 5-membered ring, and a nearby micropore. This structure optimizes the energy band structure of Fe centers and accelerates the desorption of the RDS *OH intermediate. This synergy of structural features ultimately boosts ORR electrocatalytic kinetics.

### Performance of ZAB application

**Fig. 5.**
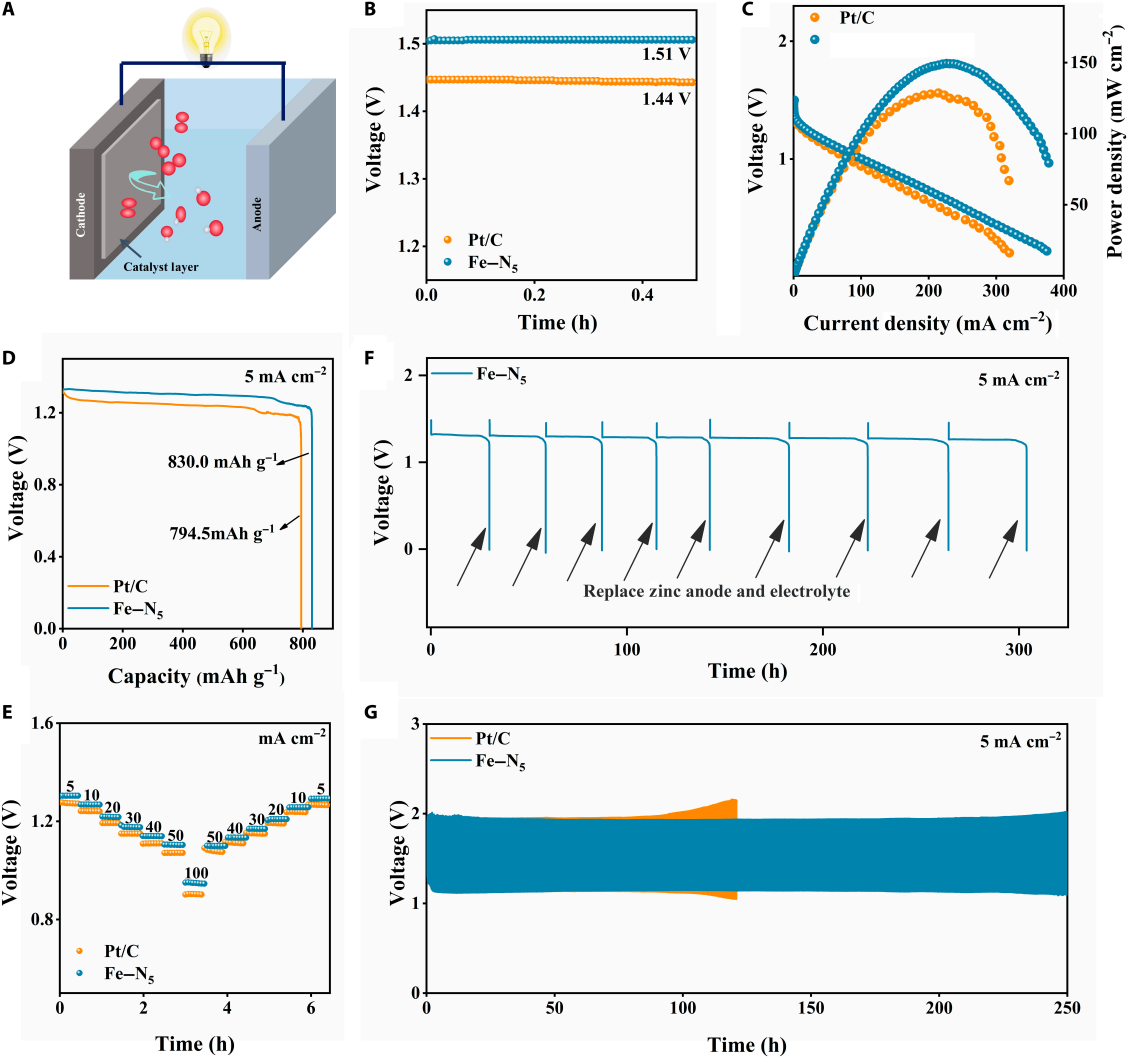
Zn–air batteries’ (ZABs’) performance. (A) Schematic of a ZAB. (B) Open-circuit voltage (OCV) of Fe–N_5_-based and Pt/C-based ZABs. (C) Polarization curves and corresponding power densities for ZABs. (D) Voltage–capacity curves at 5 mA cm^−2^. (E) Rate capability test of the ZABs with Fe–N_5_ and Pt/C. (F) Discharge curves for the ZABs with Fe–N_5_ at a current density of 5 mA cm^−2^. (G) Discharge/recharge cycling curves of rechargeable ZABs based on Fe–N_5_ + RuO_2_ and Pt/C + RuO_2_ at the current density of 5 mA cm^−2^.

Leveraging the excellent ORR properties of Fe–N_5_, a proof-of-concept ZAB experiment was conducted to demonstrate its feasibility as an air cathode (Fig. 5A and Fig. S35), of which a zinc foil was used as the anode and 6.0 M KOH + 0.2 M Zn(Ac)_2_ was applied as the electrolyte. The Fe–N_5_-based ZAB exhibits a high OCV of 1.51 V (Fig. 5B), a peak power density of 150.0 mW cm^−2^ at 228.0 mA cm^−2^ (Fig. 5C), and a specific capacity of 830.0 mAh g^−1^ at 5 mA cm^−2^ (Fig. 5D), which are higher than those of commercial Pt/C (1.44 V; 128.0 mW cm^−2^ at 212.0 mA cm^−2^; 794.5 mAh g^−1^). Additionally, the Fe–N_5_-based ZAB shows high stability with a minimal voltage fluctuation of 10 mV even when it fluctuates from 5 to 100 mA cm^−2^ (Fig. 5E), highlighting its superior rate capability. The Fe–N_5_-based ZAB exhibits only a 3.8% voltage loss after 300 h of continuous discharge at 5 mA cm^−2^ (Fig. 5F), exceeding the performance of the state-of-the-art ZAB. Of greater significance, the ZAB fabricated using Fe–N_5_ + RuO_2_ achieves ultrastable cycling (>250 h), demonstrating the viability of Fe–N_5_ for practical device implementation (Fig. 5G), which can be attributed to the strong FeN_5_ coordination configuration within Fe–N_5_ constructed by the dual-confinement effect of T-CNCs, suppressing Fe dissolution. Moreover, the Fe–N_5_-based ZAB retains round-trip efficiencies of 59.11% and 55.53% after 50 and 250 h, respectively, with an initial round-trip efficiency of 61.42%. This performance surpasses that of the ZAB fabricated with commercial Pt/C, which exhibits a more pronounced efficiency decay during operation, decreasing from an initial value of 65.28% to 58.76% at 50 h and further plummeting to 55.55% after only 100 h of cycling (Fig. S36), unequivocally verifying the exceptional long-term durability of the Fe–N_5_ catalyst and its substantial advantage over commercial Pt/C for practical energy storage applications.

## Conclusion

In summary, we successfully designed Fe–N_5_ SACs via a dual-confinement strategy that integrates spatial confinement from a wood framework and with coordination by Fe^3+^ ions. The SAC features Fe–N_5_ moieties, where each Fe center is coordinated with 4 equatorial pyridinic N atoms and 1 axial pyrrolic N atom, anchored on a N, S-codoped carbon matrix with hollow, hierarchically interconnected 3D pores. DFT calculations and XAS analyses substantiated that 3 structural features synergistically modulate adsorbate binding energies and enhance ORR kinetics: the unique Fe–N_5_ coordination environment, in situ S doping from the residual functional groups of T-CNCs, and adjacent micropores. Together, these features optimize the electronic structure of Fe active sites and facilitate the adsorption/desorption of oxygen intermediates. This work highlights confinement-effect-driven tailoring of central metal atoms within a hierarchical porous framework with atomic-level precision, offering new insights into the role of coordination environments in catalytic activity and paving the way for the rational design of efficient catalysts for practical applications in energy conversion and storage.

## Materials and Methods

### Materials

Degreasing cotton was purchased from Alibaba Taobao (Hangzhou, China). Basswood woodblocks were purchased from Alibaba Taobao (Hangzhou, China). Sodium chlorite (NaClO_2_), acetic acid (CH_3_COOH), ethanol, sulfuric acid (H_2_SO_4_, 98%), iron(III) nitrate nonahydrate (Fe(NO_3_)_3_·9H_2_O), ammonium chloride (NH_4_Cl), tertiary butanol, sodium hydroxide (NaOH), sodium chloride (NaCl), concentrated hydrochloric acid (HCl, 36% to 38%), TEMPO, sodium hypochlorite (NaClO), and sodium bromide (NaBr) were purchased from Rhawn. All reagents were of analytical grade and used without further purification. A Nafion 117 membrane was purchased from Dupont.

### Synthesis of TEMPO-modified carboxylated cellulose nanocrystals (T-CNCs)

CNCs obtained via sulfuric acid hydrolysis were used as the pristine material and denoted as S-CNCs. TEMPO-mediated oxidation was employed to synthesize carboxylated CNCs (T-CNCs), following a 2-step functionalization protocol. In the first step, surface activation of S-CNCs was performed. An aqueous suspension of S-CNCs (0.5 wt%) was mixed with hydrochloric acid (HCl, 50.0 mM) and magnetically stirred at 80 °C for 24 h. After cooling to room temperature, the suspension was partially neutralized to a pH of approximately 4.0 using sodium hydroxide (NaOH) solution. The activated S-CNCs were then purified by dialysis against deionized water to remove residual electrolytes, followed by concentration via ultrafiltration to adjust the solid content for subsequent reactions. The second step involved TEMPO-mediated oxidation of the activated S-CNCs. To the concentrated S-CNC suspension (1.0 wt% in deionized water), TEMPO (0.1 g per gram of dry S-CNCs), sodium hypochlorite (NaClO, 1.9 g per gram of dry S-CNCs), and sodium bromide (NaBr, 1.0 g per gram of dry S-CNCs) were sequentially added under stirring until complete dissolution. The reaction mixture was stirred at room temperature for 4 h, during which the pH was maintained between 10.0 and 11.0 by dropwise addition of 1.0 M NaOH. This pH range was sustained until no further pH drift was observed, indicating the completion of oxidation. Subsequently, NaCl was added to flocculate the T-CNCs, and the product was washed by centrifugation and decanting (3 times with 0.5 M NaCl, 2 times with 0.1 M HCl, and once with deionized water) before final purification by dialysis.

CNCs-H80: S-CNCs were activated with 50 mM hydrochloric acid at 80 °C for 24 h, without TEMPO oxidation; all other preparation procedures are identical to those of T-CNCs. CNCs-H100: S-CNCs were activated with 250 mM hydrochloric acid at 100 °C for 48 h, without TEMPO oxidation; all other preparation procedures are identical to those of T-CNCs. CNC-100: S-CNCs were activated with 250 mM hydrochloric acid at 100 °C for 48 h, followed by TEMPO oxidation. All other preparation procedures were consistent with those of T-CNCs.

### Synthesis of porous networks in wood frameworks

Balsa wood with dimensions of 20 mm × 20 mm × 3 mm (tangential × radial × axial) was treated with 2.0 wt% NaClO_2_ at 70 °C, maintaining a pH of 4.6 for 18 h, followed by washing with deionized water to neutrality. It was then freeze-dried to obtain delignified balsa wood.

Two pieces of the delignified balsa wood were impregnated in a 0.9 wt% T-CNC aqueous solution for 30 min followed by ultrasonication for 100 min. The T-CNC-impregnated wood and residual solution were collectively stored in a refrigeration chamber at 8 °C overnight. Subsequently, the T-CNC-loaded scaffolds were transferred into 25.0 ml of Fe(NO_3_)_3_ (0.1 M) ethanolic solution and maintained at 8 °C for 3.5 h. The treated samples were then immersed in a 20.0 wt% *tert*-butanol aqueous solution at 8 °C for 6 h. Finally, freeze-drying yielded porous architectures within the wood frameworks. The obtained product was designated as W-TCNC-Fe.

### Synthesis of Fe–N_5_

The Fe–N_5_ precursor (W-TCNC-Fe) was pre-treated under 300 °C for 2 h in a N_2_ atmosphere. The resulting material was then thoroughly ground with NH_4_Cl at a 20-fold mass ratio to form homogeneous powder, followed by immersion in 20.0 ml of deionized water and magnetically stirred at 60 °C for 24 h until complete dryness. Subsequently, the dried powder was directly heated to the target temperature (1,000 °C) at a heating rate of 5 °C min^−1^ and kept for 4 h under a flow of N_2_ in a tube furnace. At last, the Fe–N_5_ catalyst was successfully synthesized, featuring hollow-interconnected hierarchical porous architectures anchored on wood cell walls.

The synthesis procedures described above are as follows:

Fe@CW was prepared by immersing 2 delignified balsa wood pieces into 0.9 wt% S-CNC solution, followed by identical experimental steps as those for Fe–N_5_. N@TCW was synthesized by immersing the T-CNC-impregnated delignified balsa wood in ethanol (without the addition of Fe(NO_3_)_3_), with the remaining steps identical to those of Fe–N_5_. Fe@TCW was obtained by carbonizing the Fe–N_5_ precursor, followed by direct grinding without mixing with NH_4_Cl, while maintaining the same synthesis parameters as those for Fe–N_5_. Fe@W was synthesized by directly impregnating Fe(NO_3_)_3_ into the wood framework (without the T-CNC impregnation step in the delignified balsa wood), with all other procedures identical to those of Fe–N_5_. Fe@H80 was prepared by immersing 2 delignified balsa wood pieces into 0.9 wt% CNC-H80 solution, followed by identical experimental steps as those for Fe–N_5_. Fe@H100 was prepared by immersing 2 delignified balsa wood pieces into 0.9 wt% CNC-H100 solution, followed by identical experimental steps as those for Fe–N_5_. Fe–N(100) was prepared by immersing 2 delignified balsa wood pieces into 0.9 wt% CNC-100 solution, followed by identical experimental steps as those for Fe–N_5_. Moreover, the entire experimental process eliminated the tedious acid washing steps typically required in the preparation of single-atom Fe–N–C catalysts.

### Characterizations

#### Electrochemical measurements

All electrochemical measurements were performed with a CHI 760E electrochemical analyzer in a standard 3-electrode system (Shanghai Chenhua Instruments Co. Ltd, China). A Pt foil and an Ag/AgCl (saturated KCl) electrode were used as a counter electrode and a reference electrode, respectively. A glassy carbon rotating disk electrode (GC-RDE, 5 mm in diameter) was coated with catalyst ink and served as the working electrode. In order to prepare the working electrode, 5.0 mg of catalyst was added into 1.0 ml of ethanol/water (980.0 μl, *v*:*v* =1:1) and 20.0 μl of Nafion (2.0 wt%) and the above mixture was sonicated for 30 min to form a homogeneous slurry. Then, 10.0 μl of the catalyst ink was dropped onto the working electrode with a loading amount of 255.0 μg cm^−2^. For comparison, the commercially available Pt/C catalyst solution (20.0 wt%) was also prepared with a catalyst loading of 255.0 μg cm^−2^. Before the electrochemical measurements, O_2_ was purged into the electrolytic solutions for 30 min and held during the experiment to ensure O_2_ saturation in the electrolyte. CV measurements were performed in O_2_-saturated 0.1 M KOH solution at 50 mV s^−1^ in a voltage range of 0 to 1.2 V. Linear sweep voltammetry measurements were carried out in O_2_-saturated 0.1 M KOH solution at different rotating rates from 1,600 to 400 rpm with a scan rate of 5 mV s^−1^. ECSAs were evaluated by the double-layer capacitance (*C*_dl_) obtained via CV curves at 5, 10, 15, 20, 25, and 30 mV s^−1^ scan rates in a non-Faradaic range. All potentials are converted to reversible hydrogen electrode (RHE) by the Nernst equation:

$$E_{\mathrm{RHE}}=E_{\left(Ag/Ag\mathrm{Cl}\right)}+0.591\times \mathrm{pH}$$ (1)

The electron transfer number (*n*) was determined by the K-L equation:

$$\begin{array}{c} \frac{1}{J}=\frac{1}{J_{k}}+\frac{1}{J_{L}}=\frac{1}{J_{k}}+\frac{1}{B\omega^{1/2}}\\ B=0.62{\mathrm{nFC}}_{O_{2}}D_{O_{2}}^{\frac{2}{3}}v^{-\frac{1}{6}} \end{array}$$ (2)

where *J* is the measured current density, *J*_k_ is the kinetic current density, *J*_L_ is the limiting current density, *ω* is the angular velocity of the disk, *n* is the overall number of electrons transferred in oxygen reduction, *F* is the Faraday constant (96,485 C mol^−1^), *C*_O2_ is the bulk concentration of O_2_ (1.2 × 10^−6^ mol cm^−3^), *D*_O2_ is the diffusion coefficient of O_2_ in 0.1 M KOH (1.9 × 10^−5^ cm^2^ s^−1^), and *ν* is the kinematic viscosity of the electrolyte (0.01 cm^2^ s^−1^).

The RRDE test was done by using an RRDE with a Pt ring with a 6.5-mm inner diameter and an 8.5-mm outer diameter. The RRDE test was conducted in an O_2_-saturated 0.1 M KOH electrolyte at 1,600 rpm at a scan rate of 5 mV s^−1^. The peroxide species yield (H_2_O_2_%) and *n* were calculated with the following equations:

$$\begin{array}{c} \%\left(H_{2}O_{2}\right)=200\times \frac{I_{R}/N}{I_{D}+I_{R}/N}\\ n=4\times \frac{I_{D}}{I_{D}+I_{R}/N} \end{array}$$ (3)

where *I*_D_ is the disk current, *I*_R_ is the ring current, and *N* = 0.37 is the current collection efficiency of the Pt ring.

The kinetic current density (*J*_k_) was calculated from the K-L equation:

$$J_{k}=\frac{J_{L}\times J}{J_{L}-J}$$ (4)

The TOFs at 0.85 and 0.90 V for SACs were calculated from the equations

$$\begin{array}{l} \mathrm{TOF}=\frac{J_{k}\times S}{4\times F\times N}\\ N=W\times \frac{m}{M} \end{array}$$ (5)

where *J*_k_ is the kinetic current at 0.90 and 0.85 V, *S* is the disk area of the rotating disk electrode (0.196 cm^2^), *N* is the number of active sites, *W* is the metal content of the catalyst measured by ICP-MS, *m* is the loading of the catalyst on the electrode, and *M* is the relative atomic mass.

The quantitative analysis of active sites was conducted using the nitrite stripping methodology developed by Kucernak’s research group [1]. The CV profiles of the catalyst were recorded in nitrogen-saturated 0.1 M KOH electrolyte at a constant scan rate of 10 mV s^−1^. Following selective poisoning of catalytic active sites with nitrite species, the dynamic evolution of CV curves was continuously monitored until complete restoration of their original profiles was achieved. Electrochemical characterization was conducted within a potential window ranging from 0.4 to −0.3 V vs. RHE.

$$\mathrm{SD}\;\left(\text{sites}\;g^{-1}\right)=\frac{Q_{\text{strip}}\times N_{A}}{n_{\text{strip}}\times F}$$ (6)

$$\mathrm{TOF}=\frac{J_{k}\times N_{A}}{\mathrm{SD}\times F}$$ (7)

where *Q*_strip_ is the coulometric charge in units of coulomb associated with the NO stripping peak, *N*_A_ is Avogadro’s constant (6.022 × 10^23^ mol^−1^), *n*_strip_ is the number of electrons associated with the reduction of one adsorbed nitrosyl per site and its value is 5, *F* is the Faraday constant (96,485 C mol^−1^), and *J*_k_ is the current at 0.95 V.

MA is calculated by

$$\mathrm{MA}=\frac{J_{k}}{M_{\text{metal}}}$$ (8)

where *M*_metal_ is the metal loading mass (g) of electrocatalysts. The metal contents were determined by ICP-MS.

#### ZAB assembly and measurement

ZABs were fabricated with a 2-electrode configuration to evaluate the practical application potential of Fe–N_5_. A polished zinc (Zn) foil (thickness: 0.3 or 0.5 mm; geometric area: 2 cm^2^) served as the anode, while carbon paper (1 cm^2^) loaded with catalysts functioned as the air cathode, with the catalyst loading protocols adjusted based on specific performance tests to ensure targeted and comparable characterizations. For rechargeable cycling tests, a mixed catalyst ink was prepared using Fe–N_5_ (as the ORR catalyst) and ruthenium dioxide (RuO_2_, as the oxygen evolution reaction catalyst). This ink was uniformly dropped onto the carbon paper, with the mass loading of Fe–N_5_ strictly controlled at 1.0 mg cm^−2^. The electrolyte—composed of 6.0 M KOH and 0.2 M Zn(Ac)_2_—was circulated between the anode and cathode compartments using a small peristaltic pump, which ensured continuous ion transport and uniform electrolyte distribution to maintain stable electrochemical performance during repeated charge–discharge cycles. For OCV, peak power density, and specific capacity tests, a pure Fe–N_5_ catalyst (1.0 mg) was dropped onto carbon paper (1 cm^2^) to achieve an Fe–N_5_ mass loading of 1.0 mg cm^−2^—consistent with the cycling test to establish comparable performance benchmarks. The same 6.0 M KOH + 0.2 M Zn(Ac)_2_ mixed solution was used as the electrolyte for these steady-state characterizations. The specific capacity (C, mAh g^−1^) of the ZABs was calculated using the applied discharge current (*I*, mA), total discharge duration (*t*, h), and the mass of Zn consumed during discharge (*m*_Zn_, g).

$$C_{\mathrm{mAh}\;g^{-1}}=\frac{I\times t}{m_{\mathrm{Zn}}}$$ (9)

#### Material characterization

The morphology of the catalysts was monitored by TEM, and EDX mapping images were captured using FEI Talos F200X G2 (USA) equipped with an energy-dispersive x-ray spectrometer, operating at 200 kV. Scanning electron microscopy images were obtained using Zeiss Supra 55 (Germany). Fourier transform infrared spectroscopy was performed using a Thermo Fisher Scientific Nicolet iS20 spectrometer (USA). ICP analysis was carried out with Agilent 7800(MS) (USA) to measure the metal content. Powder XRD patterns were recorded using a Bruker D8 Advance x-ray powder diffractometer with Cu Kα radiation (Germany). Raman spectroscopy was performed using Horiba LabRAM HR Evolution from Japan. XPS measurements were performed on Thermo Scientific K-Alpha with Al Kα radiation source (USA). The N_2_ adsorption/desorption curves were tested at 77.3 K using a Micromeritics ASAP 2460 surface area analyzer (USA). AC HAADF-STEM characterization was performed using the Titan G2 60-300 field emission transmission electron microscope from FEI (The Netherlands). Electron paramagnetic resonance was tested using German Bruker EMXplus-6/1. The x-ray absorption fine structure (XAFS) spectra including XANES and EXAFS of the Fe K-edge were obtained at the Shanghai Synchrotron Radiation Facility at 2.5 GeV with a current of 250 mA. The metal foil was measured for energy calibration. All spectra were collected in fluorescence mode. XAS data reduction and analysis were processed by the Athena software.

### DFT calculation

All calculations were performed using DFT as implemented in the Vienna Ab initio Simulation Package with the Perdew–Burke–Ernzerhof exchange-correlation functional [2]. The electron–ion interaction was addressed using the projector-augmented-wave method and a plane wave with a cutoff energy of 450 eV. Structural models were optimized until Hellmann–Feynman forces converged below 0.02 eV/Å and energy changes were less than 10^−5^ eV. Dispersion interactions between atoms in adsorption models were accounted for using Grimme’s DFT-D3 method. A gamma-centered *k*-point mesh of 2 × 2 × 1 was adopted. A vacuum space of 12 Å was imposed along the *z*-direction.

## Data Availability

Data will be made available on request.

## References

[1] Xie X, Wei Y, Ma H, Zou P, Xiang W, Lv X, Sun P, Sun X. Electronic regulation of Fe–N_5_ sites via Zn coordination for high-efficiency oxygen reduction and rechargeable zinc-air batteries. J Colloid Interface Sci. 2026;712: Article 140107. 41707503 10.1016/j.jcis.2026.140107

[2] Zhou H, Wang Q, Zhang B, Li J, Ou T, Zhai H-J, Zhang J. Single-atom iron-decorated hierarchically porous carbon nanofibers with carbon vacancy-enhanced performance for zinc-air batteries and supercapacitors. Chem Eng J. 2025;522: Article 167822.

[3] He Y, Liu S, Priest C, Shi Q, Wu G. Atomically dispersed metal–nitrogen–carbon catalysts for fuel cells: Advances in catalyst design, electrode performance, and durability improvement. Chem Soc Rev. 2020;49:3484–3524. 32342064 10.1039/c9cs00903e

[4] Lin X, Xing H, Yu J, Zhang D, Lu X, Zhang Y, Sun Y, Zhang S, Deng C. Entropy-driven design of non-metallic heteroatoms codoped hollow carbon nanocages for superior oxygen electrocatalysis. J Colloid Interface Sci. 2026;710: Article 139990. 41633060 10.1016/j.jcis.2026.139990

[5] Zhao Y, Chen H-C, Ma X, Li J, Yuan Q, Zhang P, Wang M, Li J, Li M, Wang S, et al. Vacancy defects inductive effect of asymmetrically coordinated single-atom Fe–N_3_S_1_ active sites for robust electrocatalytic oxygen reduction with high turnover frequency and mass activity. Adv Mater. 2024;36(11):2308243. 10.1002/adma.202308243 38102967

[6] Lee SH, Kim J, Chung DY, Yoo JM, Lee HS, Kim MJ, Mun BS, Kwon SG, Sung YE, Hyeon T. Design principle of Fe–N–C electrocatalysts: How to optimize multimodal porous structures? J Am Chem Soc. 2019;141(5):2035–2045. 30620877 10.1021/jacs.8b11129

[7] Cao G, Zhao W, Han L, Teng Y, Xu S, Nguyen H, Tam KC. Enhancing droplet spreading on a hydrophobic plant surface by surfactant/cellulose nanocrystal complexes. ACS Nano. 2025;19:3549–3561. 39817302 10.1021/acsnano.4c13542

[8] Raghuwanshi VS, Garnier G. Nanoparticle decorated cellulose nanocrystals (CNC) composites for energy, catalysis, and biomedical applications. Adv Funct Mater. 2024;35(2):2412869.

[9] Zhang K, Fan C, Wang Y, Liu L, Wang X, Yang C, Li N, Puangsin B, Li J, E-kobon T, et al. Mechanical/thermomechanical–electromagnetic multifunctional cellulose nanofibril-MXene aerogel-based metamaterials. Research. 2025;8:0900. 41230109 10.34133/research.0900 PMC12604525

[10] Liu YJ, Cao WT, Ma MG, Wan P. Ultrasensitive wearable soft strain sensors of conductive, self-healing, and elastic hydrogels with synergistic “soft and hard” hybrid networks. ACS Appl Mater Interfaces. 2017;9(30):25559–25570. 28696658 10.1021/acsami.7b07639

[11] ElBachraoui F, Aymé-Perrot D, Girault HH. Ionic transport aspects of water electrolysis in alkaline media. Research. 2025;8: Article 0788. 40896399 10.34133/research.0788 PMC12393796

[12] Lin Y, Wang S, Huang J, Chen L, Bi T, Qi L, Cai Z, Zeng X, Hu P, Chen W, et al. Coupling of mechanical, self-healing, adhesion, and high-ion conducting properties in anti-freezing hydrogel electrolytes of zinc ion batteries via Fe^3+^-carboxylate coordination. Adv Funct Mater. 2025;35(37):202504726.

[13] Baretta R, Davidson-Rozenfeld G, Gutkin V, Frasconi M, Willner I. Chemical and photochemical-driven dissipative Fe^3+^/Fe^2+^-ion cross-linked carboxymethyl cellulose gels operating under aerobic conditions: Applications for transient controlled release and mechanical actuation. J Am Chem Soc. 2024;146(14):9957–9966. 38547022 10.1021/jacs.4c00625 PMC11009950

[14] Kang J, Hu C, Liu X, Zhou H, Lin X, Gu J. One-pot synthesis of magnetic nanocellulose/Fe_3_O_4_ hybrids using FeCl_3_ as cellulose hydrolytic medium and Fe_3_O_4_ precursor. ACS Sustain Chem Eng. 2024;12:5917–5926.

[15] Wang W, Pan Z, Zhang Z, Zhu Y, Shen J. Carbon aerogels loaded with noble metal nanocrystal electrocatalysts for efficient full water splitting. ACS Appl Nano Mater. 2023;6:12150–12158.

[16] Zhu Y, Zhu Z, Li H, Li S, Zhai Y, Xu S-W, Wu S, Chen Y, Wang Y, Ren R, et al. Confinement effect and hydrogen species modulation toward enhanced electrochemical CO_2_ reduction to ethanol. Research. 2025;8: Article 0796. 40746826 10.34133/research.0796 PMC12311302

[17] Pei Z, Zhang H, Guo Y, Luan D, Gu X, Lou XW. Atomically dispersed Fe sites regulated by adjacent single co atoms anchored on N-P co-doped carbon structures for highly efficient oxygen reduction reaction. Adv Mater. 2023;36(17):202306047. 10.1002/adma.202306047 37496431

[18] Khan MA, Jian C, Javed R, Ye D, Zhao H. Heteroatom sulfur-doping in single-atom Fe–N–C catalysts for durable oxygen reduction performance in zinc-air batteries. J Colloid Interface Sci. 2025;685:1077–1086. 39884095 10.1016/j.jcis.2025.01.064

[19] Parker RM, Frka-Petesic B, Guidetti G, Kamita G, Consani G, Abell C, Vignolini S. Hierarchical self-assembly of cellulose nanocrystals in a confined geometry. ACS Nano. 2016;10(9):8443–8449. 27564644 10.1021/acsnano.6b03355 PMC5043420

[20] Ellebracht NC, Jones CW. Optimized cellulose nanocrystal organocatalysts outperform silica-supported analogues: Cooperativity, selectivity, and bifunctionality in acid–base aldol condensation reactions. ACS Catal. 2019;9:3266–3277.

[21] Smith KB, Tisserant JN, Assenza S, Arcari M, Nyström G, Mezzenga R. Confinement-induced ordering and self-folding of cellulose nanofibrils. Adv Sci. 2018;6(4):201801540. 10.1002/advs.201801540 PMC6382315 30828528

[22] Wu H, Qin J, Hua X, Wang Z, Zhang Z, Zhang J. Self-assembly behavior and adhesive performance of imidazolium cation grafted cellulose nanocrystals in confined space. Carbohydr Polym. 2024;336: Article 122127. 38670758 10.1016/j.carbpol.2024.122127

[23] Garemark J, Perea-Buceta JE, Felhofer M, Chen B, Cortes Ruiz MF, Sapouna I, Gierlinger N, Kilpeläinen IA, Berglund LA, Li Y. Strong, shape-memory lignocellulosic aerogel via wood cell wall nanoscale reassembly. ACS Nano. 2023;17:4775–4789. 36716432 10.1021/acsnano.2c11220 PMC10018770

[24] Zhang P, Chen HC, Zhu H, Chen K, Li T, Zhao Y, Li J, Hu R, Huang S, Zhu W, et al. Inter-site structural heterogeneity induction of single atom Fe catalysts for robust oxygen reduction. Nat Commun. 2024;15:2062. 38453927 10.1038/s41467-024-46389-3 PMC10920901

[25] Jiang R, Li L, Sheng T, Hu G, Chen Y, Wang L. Edge-site engineering of atomically dispersed Fe–N_4_ by selective C–N bond cleavage for enhanced oxygen reduction reaction activities. J Am Chem Soc. 2018;140:11594–11598. 30168714 10.1021/jacs.8b07294

[26] Tian H, Song A, Zhang P, Sun K, Wang J, Sun B, Fan Q, Shao G, Chen C, Liu H, et al. High durability of Fe–N–C single-atom catalysts with carbon vacancies toward the oxygen reduction reaction in alkaline media. Adv Mater. 2023;35(14):202210714. 10.1002/adma.202210714 36630970

[27] Sui R, Liu B, Chen C, Tan X, He C, Xin D, Chen B, Xu Z, Li J, Chen W, et al. Constructing asymmetric Fe–Nb diatomic sites to enhance ORR activity and durability. J Am Chem Soc. 2024;146:26442–26453. 39267445 10.1021/jacs.4c09642

[28] Liu S, Li C, Zachman MJ, Zeng Y, Yu H, Li B, Wang M, Braaten J, Liu J, Meyer HM III, et al. Atomically dispersed iron sites with a nitrogen–carbon coating as highly active and durable oxygen reduction catalysts for fuel cells. Nat Energy. 2022;7:652–663.

[29] Xu Y, Ma Y, Chen X, Wu K, Wang K, Shen Y, Liu S, Gao XJ, Zhang Y. Regulating reactive oxygen intermediates of Fe−N−C SAzyme via second-shell coordination for selective aerobic oxidation reactions. Angew Chem Int Ed Engl. 2024;63(36):202408935. 10.1002/anie.202408935 38895986

[30] Barrio J, Pedersen A, Sarma SC, Bagger A, Gong M, Favero S, Zhao CX, Garcia-Serres R, Li AY, Zhang Q, et al. FeNC oxygen reduction electrocatalyst with high utilization penta-coordinated sites. Adv Mater. 2023;35(14):202211022. 10.1002/adma.202211022 36739474

[31] Xu B, Li S, Zheng L, Liu Y, Han A, Zhang J, Huang Z, Xie H, Fan K, Gao L, et al. A bioinspired five-coordinated single-atom iron nanozyme for tumor catalytic therapy. Adv Mater. 2022;34:202107088. 10.1002/adma.202107088 35102632

[32] Mun Y, Lee S, Kim K, Kim S, Lee S, Han JW, Lee J. Versatile strategy for tuning ORR activity of a single Fe–N_4_ site by controlling electron-withdrawing/donating properties of a carbon plane. J Am Chem Soc. 2019;141:6254–6262. 30920818 10.1021/jacs.8b13543

