# Supplementary material for: Dual-Confinement Strategy Enables Highly Efficient Oxygen Reduction with Fe–N5 Electrocatalysts
Source: Research (Wash D C). 2026 Jun 17;9:1333. doi: 10.34133/research.1333 (PMC13273148; doi:10.34133/research.1333)
Supplement: Supplementary 1 — Figs. S1 to S36 Tables S1 to S9 [file research.1333.f1.pdf]

Supporting Information for

Dual-Confinement Strategy Enables Highly Efficient Oxygen Reduction with Fe–N$_5$ Electrocatalysts

Shilei Li[a, c‡], Jingshuo Liu[a, c ‡], Zhihang Liu[a, c], Congcong Yang[a, c], Jian Li[a, c], Zhiqun Lin[b], Likun Gao[a, c *]*

a. State Key Laboratory of Woody Oil Resources Utilization, Northeast Forestry University, Harbin, 150040, PR China

b. Department of Chemical and Biomolecular Engineering, National University of Singapore, Singapore 117585

c. Key Laboratory of Bio-based Material Science & Technology, Ministry of Education, Northeast Forestry University, Harbin 150040, PR China

*Corresponding author: Likun Gao (gaolk@nefu.edu.cn) and Zhiqun Lin (z.lin@nus.edu.sg).

‡ These authors contributed equally.

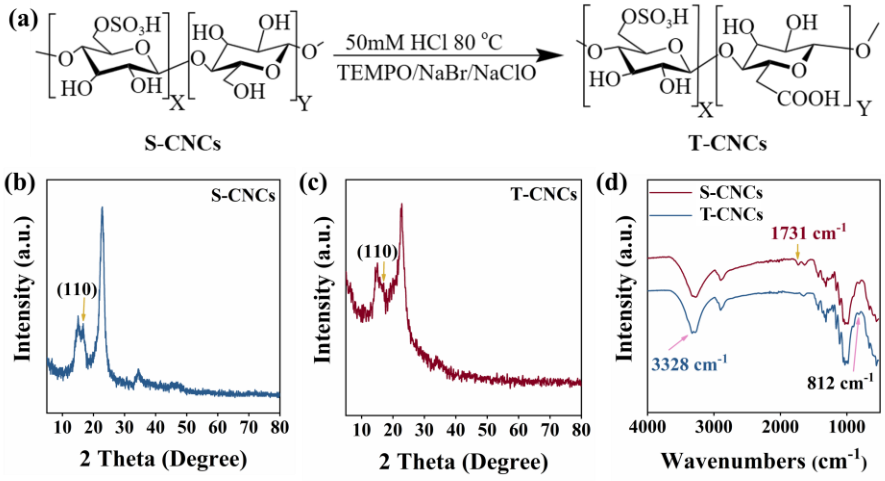

Figure S1 The carboxylation process of S-CNCs.

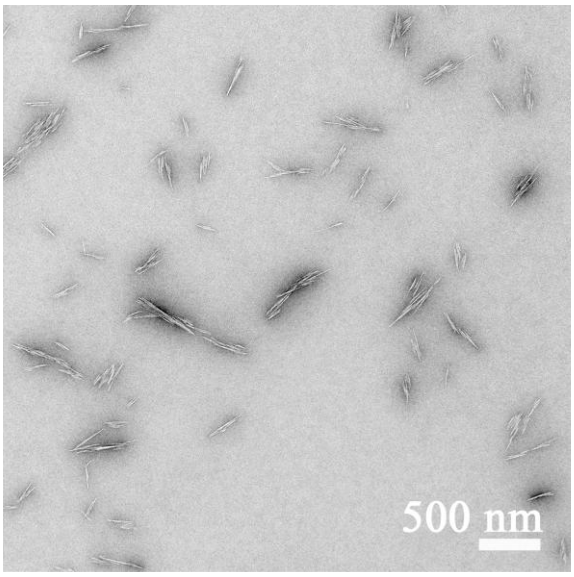

Figure S2 TEM image of S-CNCs.

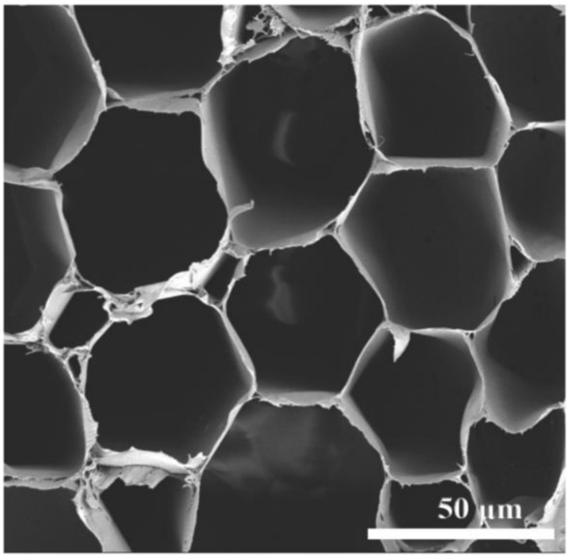

Figure S3 SEM image of delignified wood.

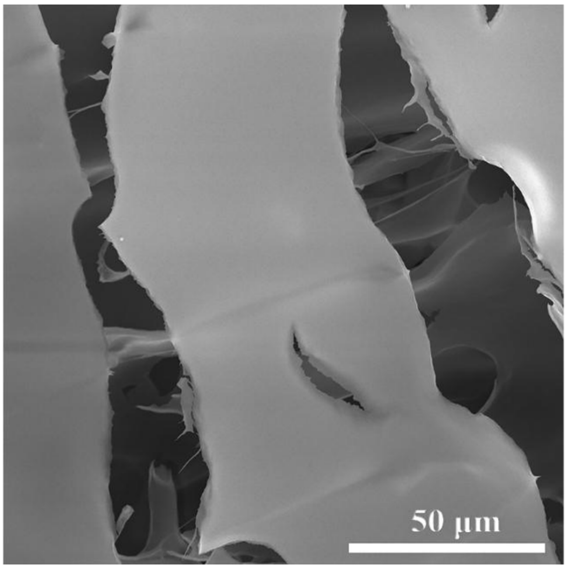

Figure S4 SEM image of T-CNCs that self-assembled without both wood framework and Fe^{3+} ions.

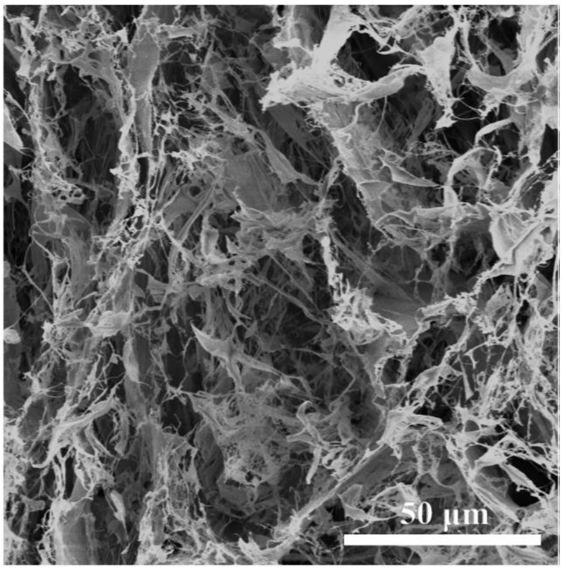

Figure S5 SEM image of T-CNCs that self-assembled with wood framework but without Fe^{3+} ions.

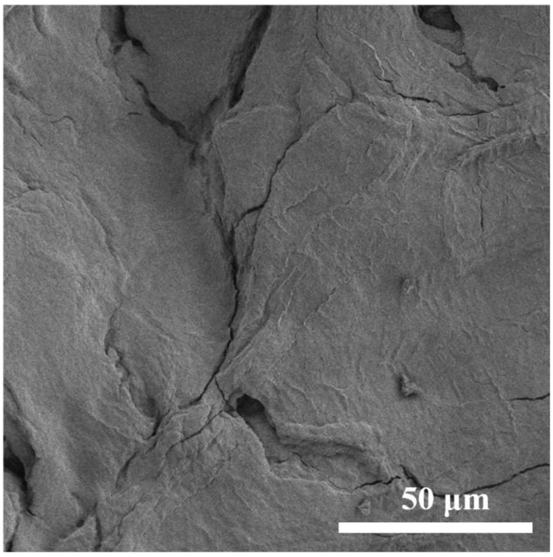

Figure S6 SEM images of T-CNCs that self-assembled with Fe^{3+} ions but without wood framework.

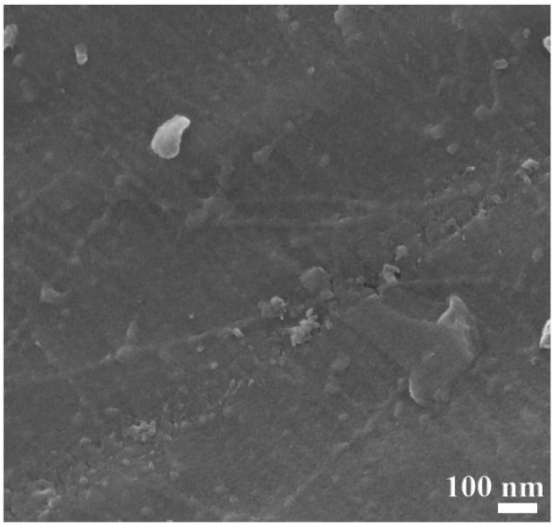

Figure S7 SEM image of Fe@W.

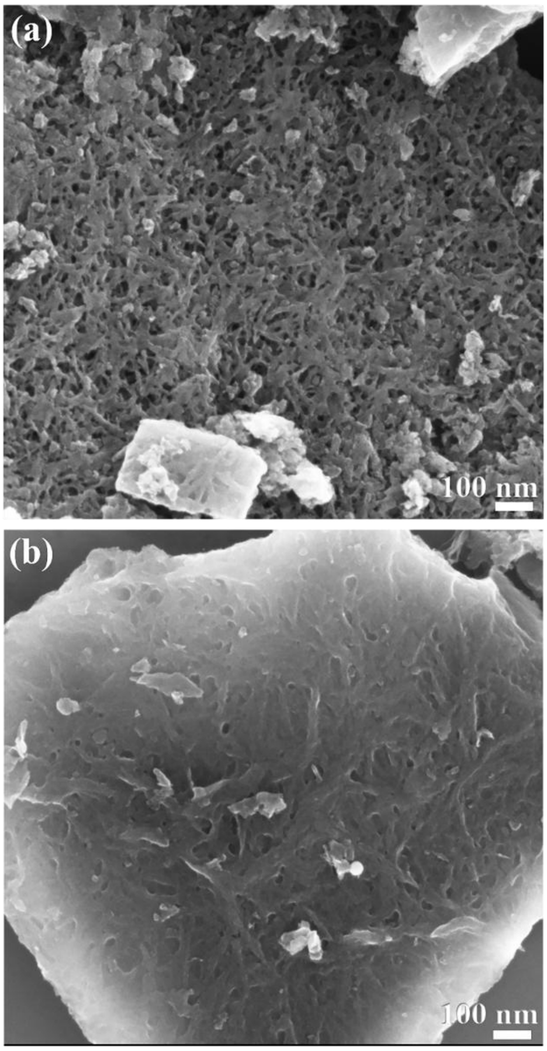

Figure S8 SEM images of (a) Fe@TCW and (b) N@TCW.

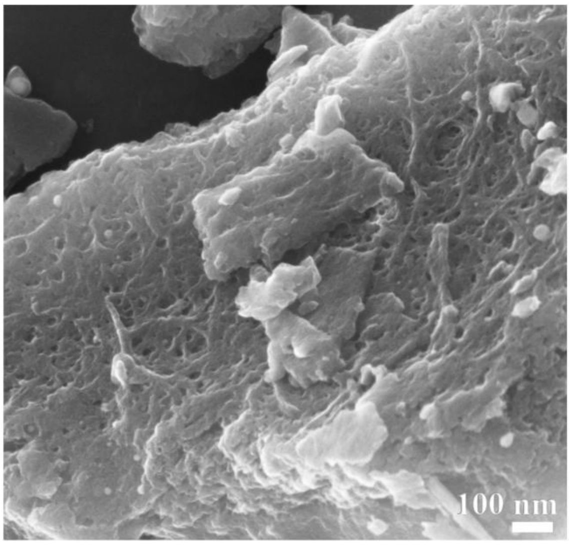

Figure S9 SEM image of Fe@CW.

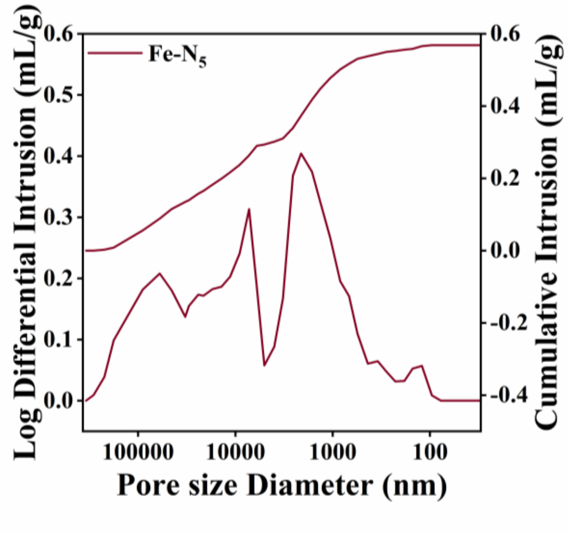

Figure S10 Mercury intrusion porosimetry (MIP) curves of Fe–N_5: differential pore-size distribution and cumulative intrusion.

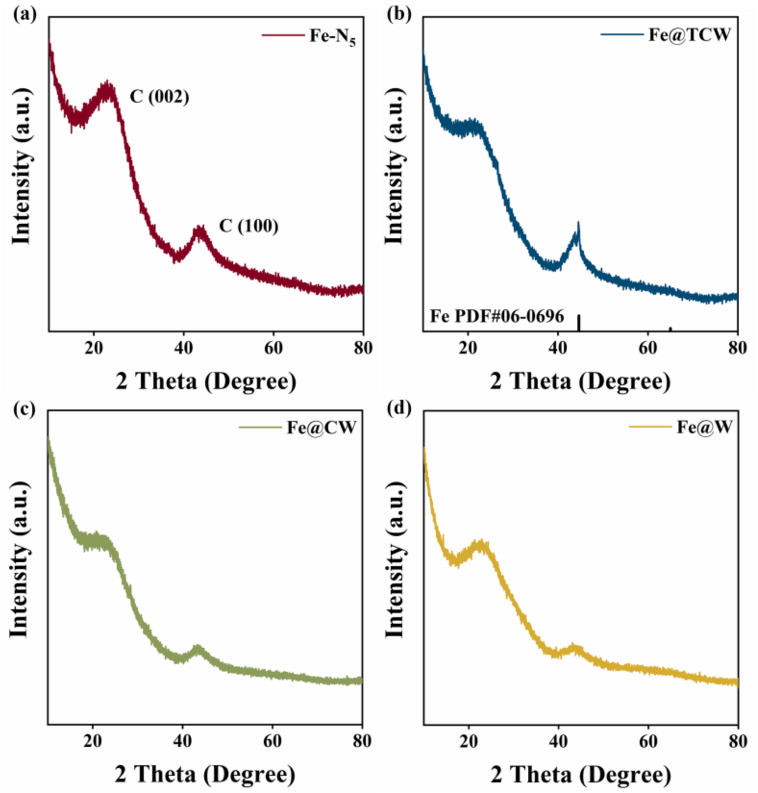

Figure S11 XRD patterns of (a) Fe–N_5, (b) Fe@TCW, (c) Fe@CW, and (d) Fe@W.

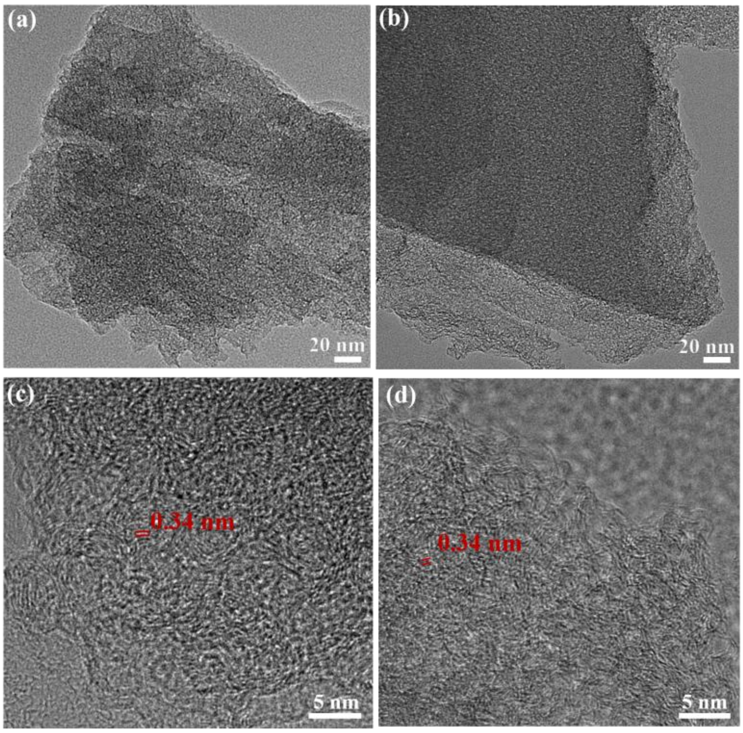

Figure S12 TEM images of (a) Fe@CW and (b) Fe@W and HRTEM images of (c) Fe@CW and (d) Fe@W.

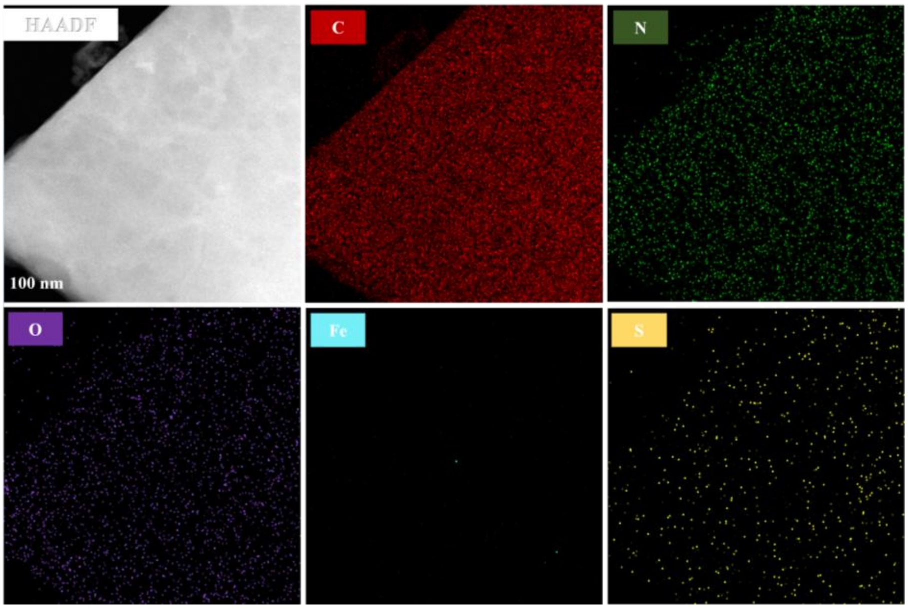

Figure S13 HAADF-STEM image and corresponding EDS elemental mapping of C, N, O, Fe, and S of Fe@CW.

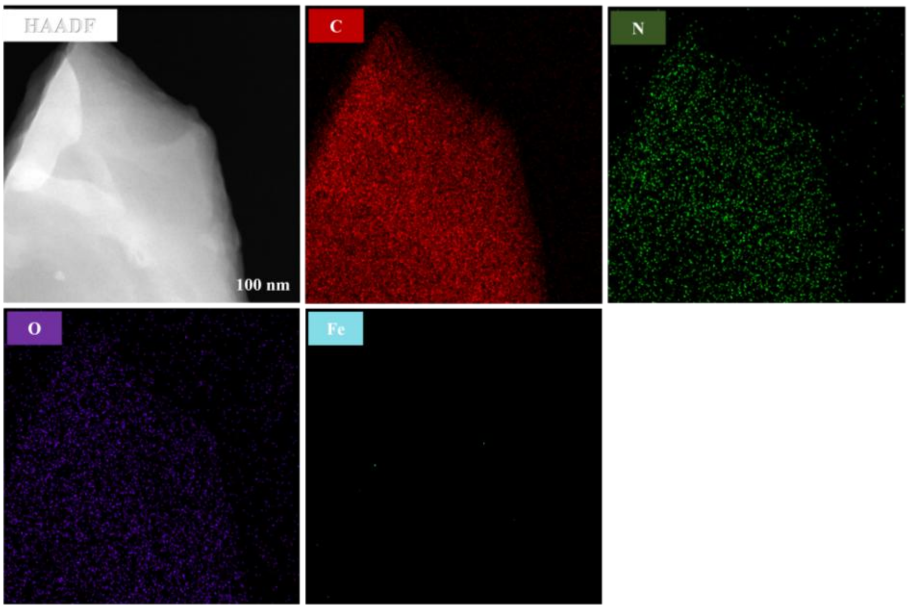

Figure S14 HAADF-STEM image and corresponding EDS elemental mapping of C, N, O, and Fe of Fe@W.

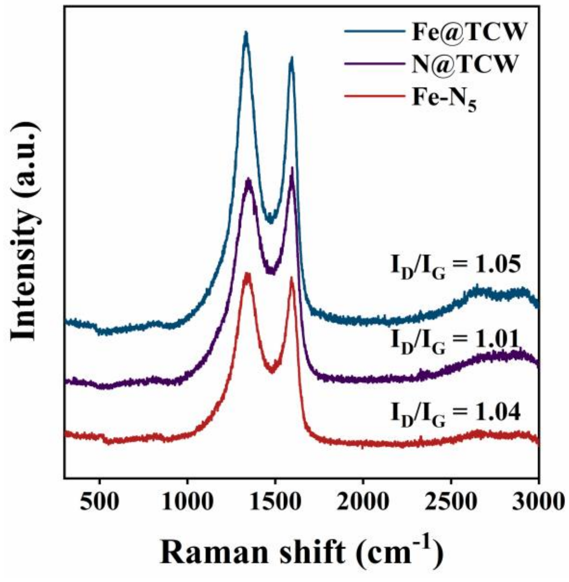

Figure S15 Raman spectroscopies of Fe@TCW, N@TCW, and Fe–N_5.

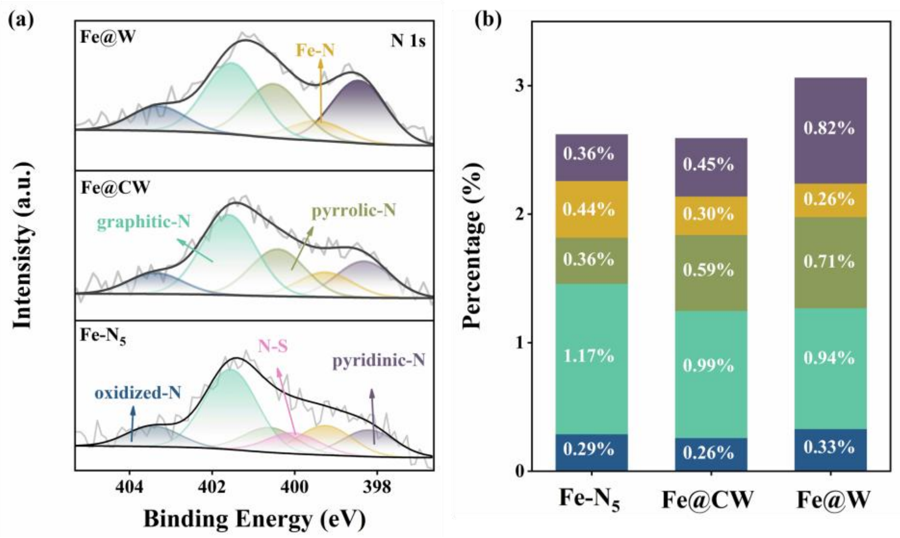

Figure S16 (a) High-resolution N 1s XPS spectra of Fe–N_5, Fe@CW, and Fe@W and (b) corresponding proportions of N species. Dark purple: pyridinic N. Gold: Fe–N. Olive green: pyrrolic N. Cyan green: graphitic N. Deep cyan: oxidized N. Pink: N–S.

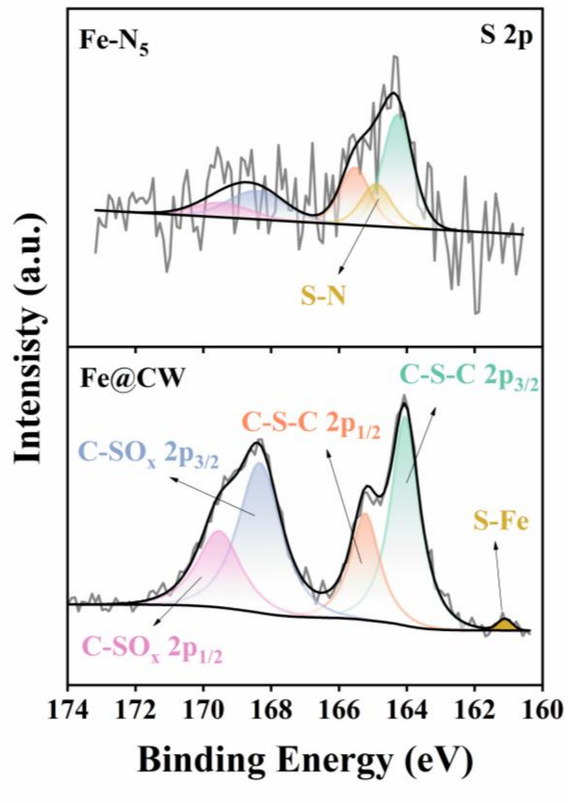

Figure S17 High-resolution S 2p XPS spectra of Fe–N_5 and Fe@CW.

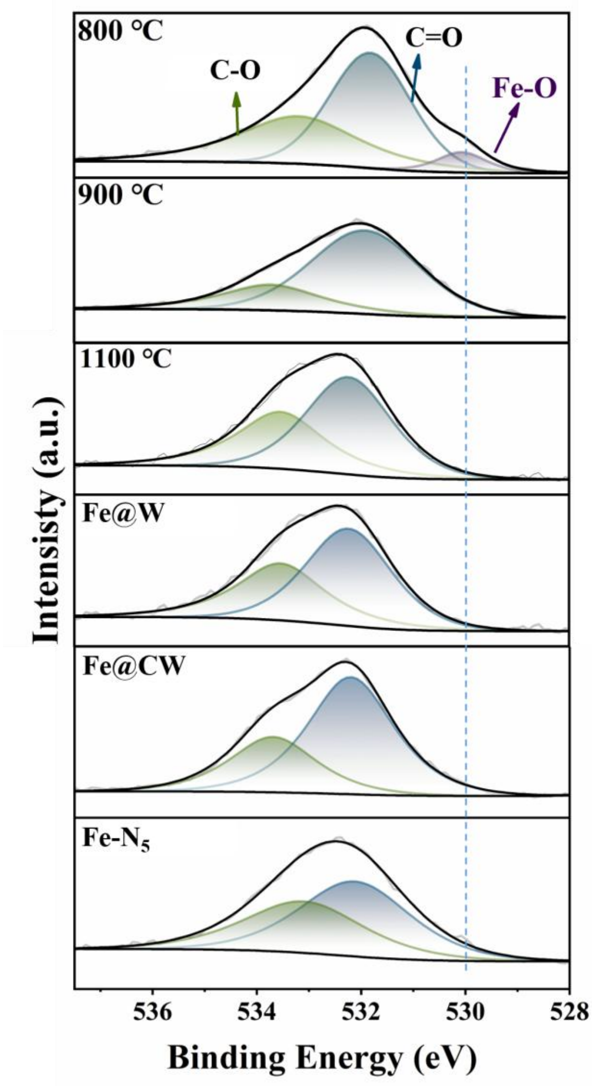

Figure S18 High-resolution O 1s XPS spectra of 800ºC, 900ºC, 1100ºC, Fe@W, Fe@CW, and Fe–N_5.

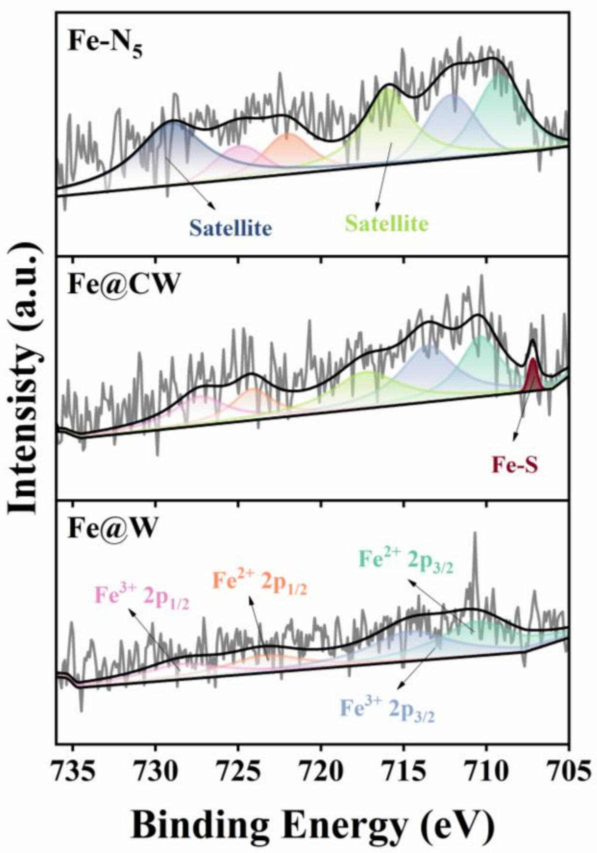

Figure S19 High-resolution Fe 2p XPS spectra of Fe–N_5, Fe@CW, and Fe@W.

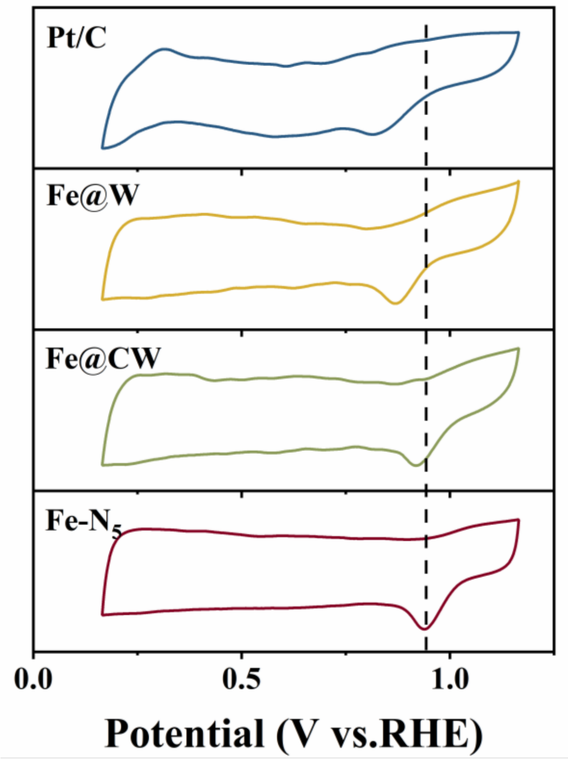

Figure S20 CV curves for Pt/C, Fe@W, Fe@CW, and Fe–N_5 recorded in O_2-saturated 0.1 M KOH with a scan rate of 50 mV s^{-1} without rotation.

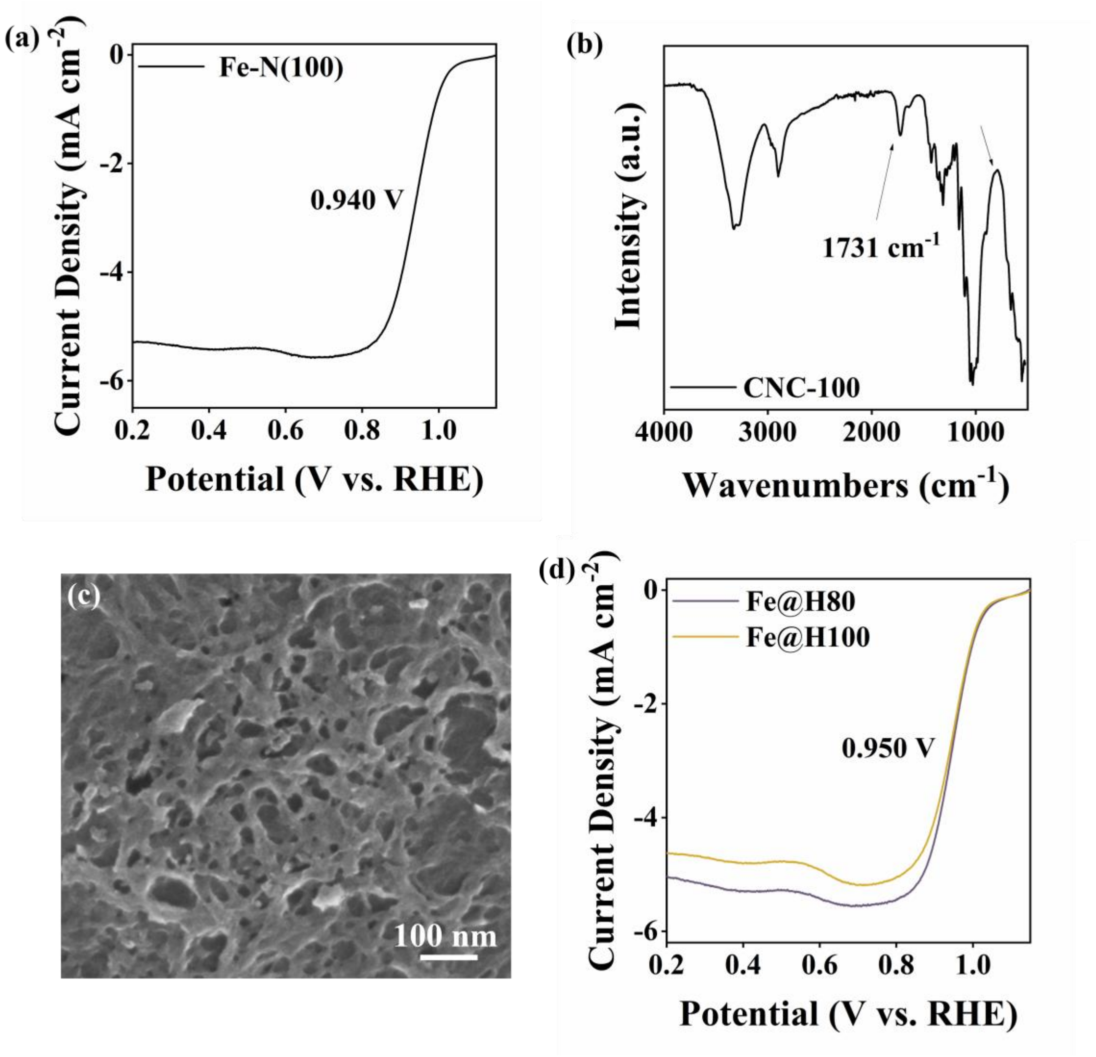

Figure S21 (a) LSV curve ($E_{1/2}$ of 0.940 V and E_{onset} of 1.080 V) of Fe–N(100) (b) FTIR spectroscopy (no peak at 812 cm^{-1} corresponding to –OSO_3H groups) of CNC-100 (c) SEM image of of Fe–N(100) (d) LSV curves ($E_{1/2}$ of 0.950 V) of Fe@H80 and Fe@H100.

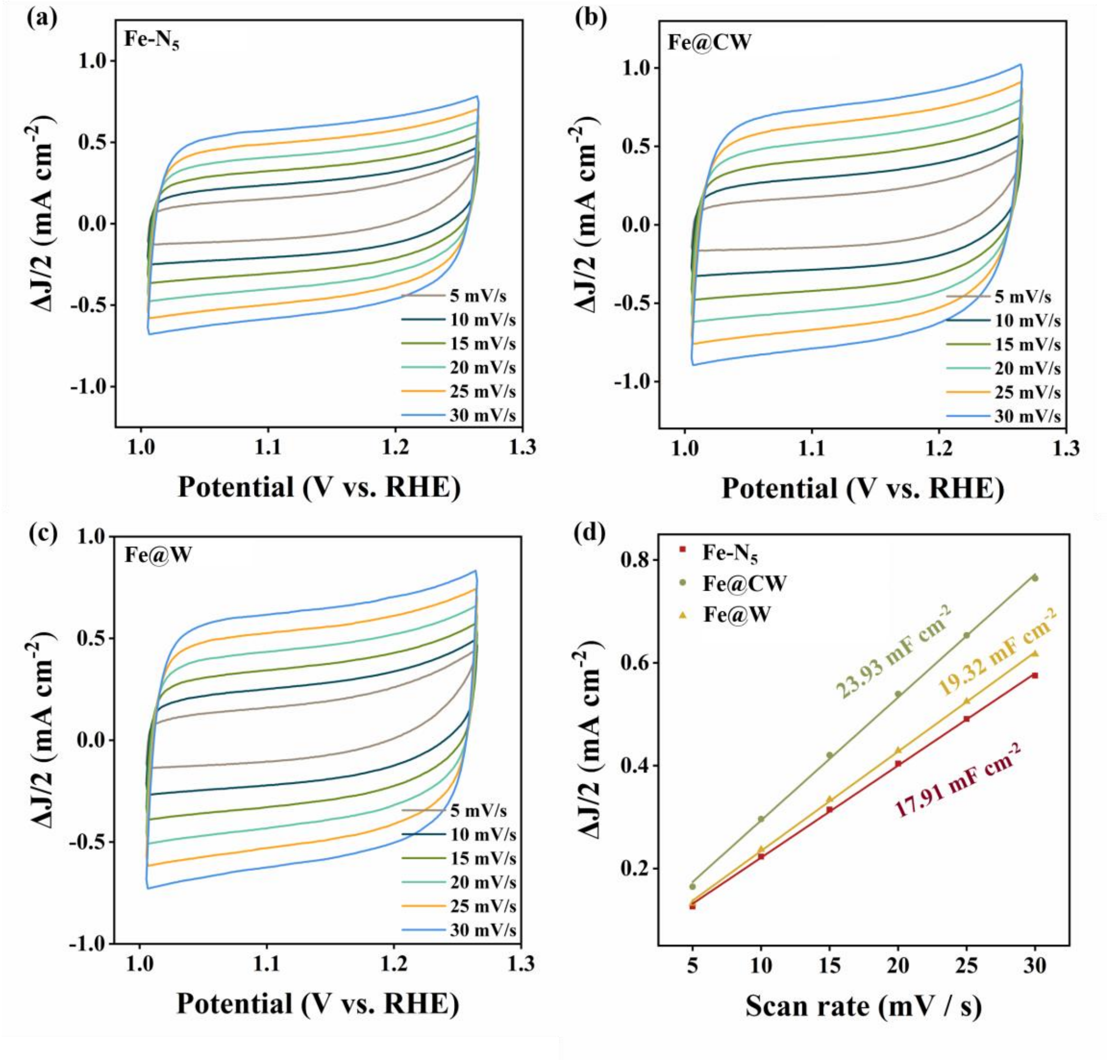

Figure S22 CV curves at different scan rates and double-layer capacitance (C_{dl}) of prepared catalysts. (a) CV for Fe–N_5, (b) CV for Fe@CW, (c) CV for Fe@W, and (d) double-layer capacitance. The C_{dl} is in proportion to electrochemical active surface area (ECSA).

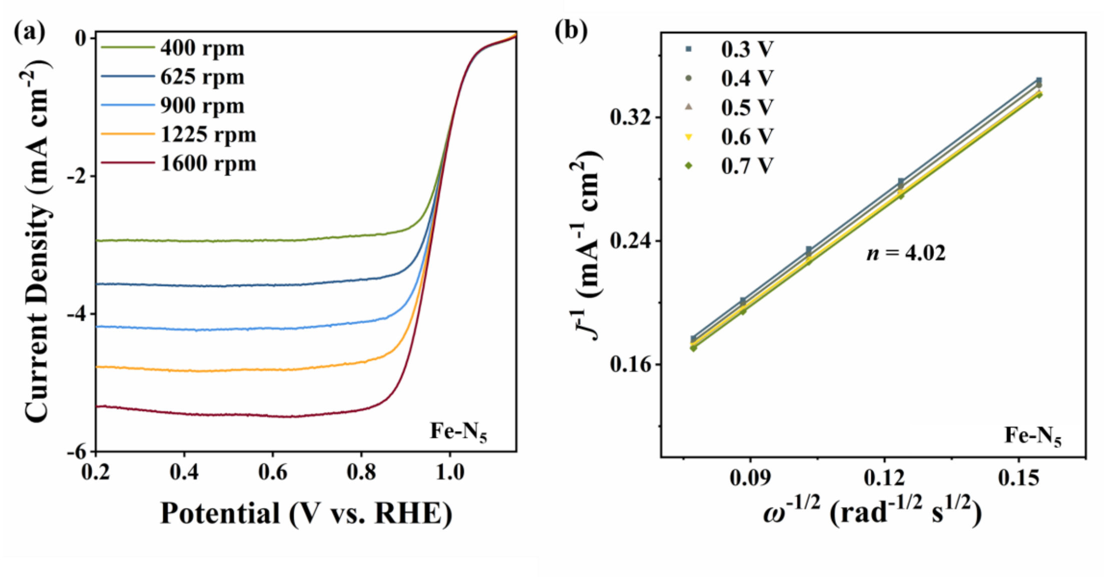

Figure S23 (a) ORR polarization curves at different rotation speeds in 0.1 M KOH and (b) The K-L plots at different potentials including *n* of Fe–N_5.

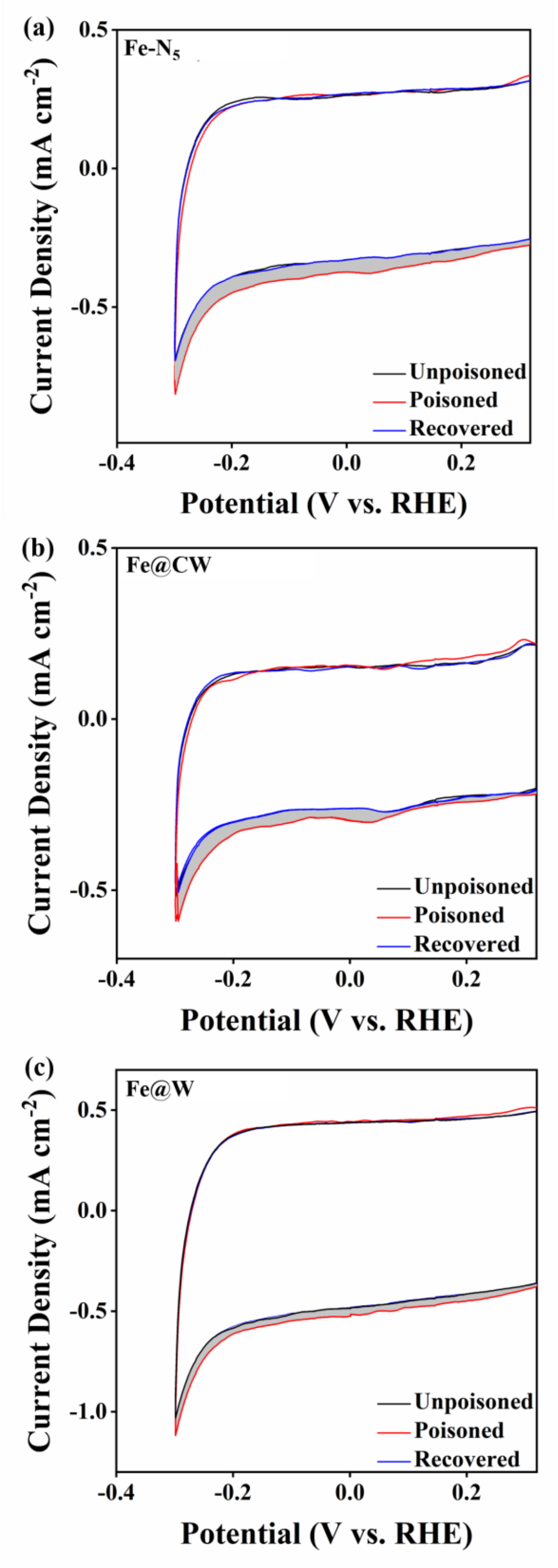

Figure S24 CV curves for (a) Fe–N_5, (b) Fe@CW, and (c) Fe@W at unpoisoned, poisoned and recovered stages of the nitrite stripping protocol, respectively.

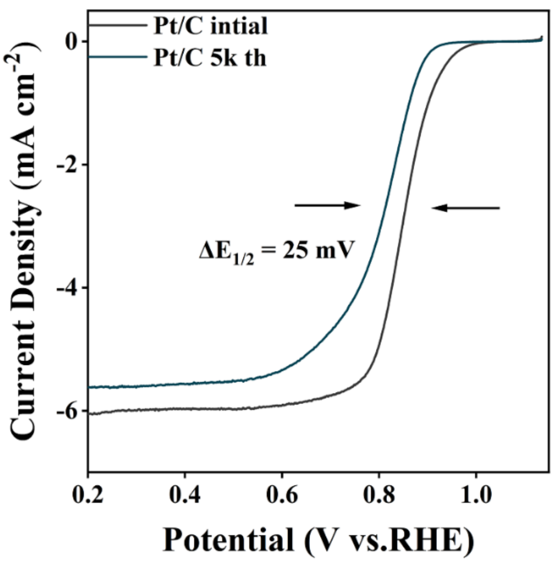

Figure S25 ORR polarization curves before and after 5000 cycles of the Pt/C in 0.1 M KOH.

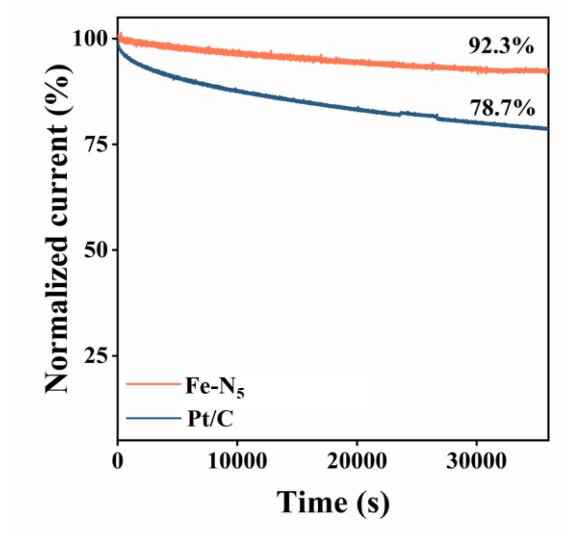

Figure S26 relative retention *i*-t curves for Fe–N_5 and Pt/C that measured in 0.1 M KOH.

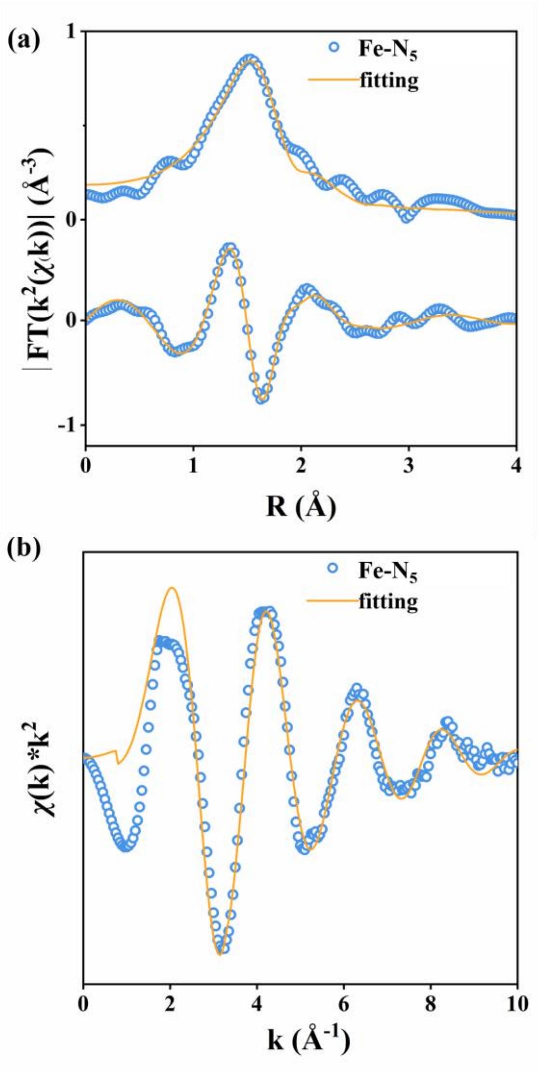

Figure S27 Fe K-edge EXAFS analysis of Fe–N_5 in (a) R space and (b) k space.

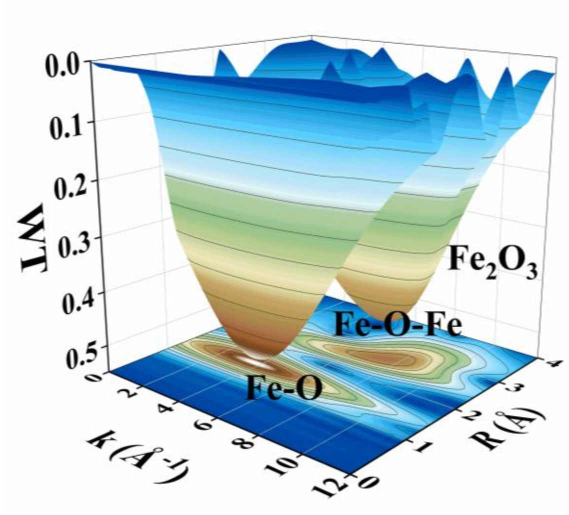

Figure S28 WT-EXAFS of Fe K-edge for Fe_2O_3.

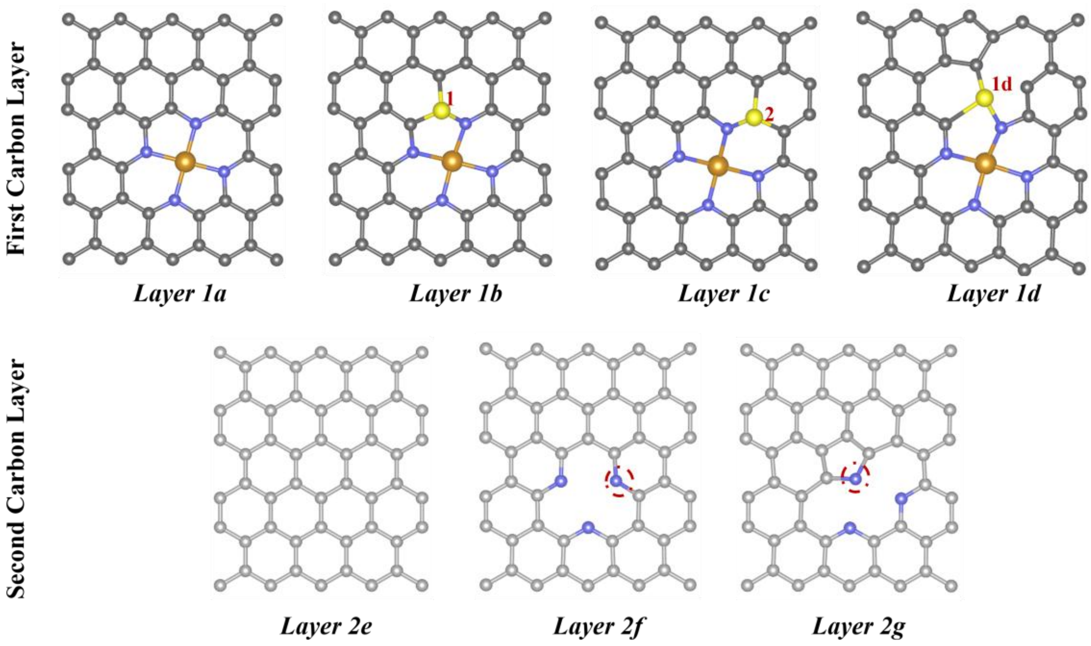

Figure S29 Illustration of proposed various structures. Dark gray: first carbon layer. Light gray: second carbon layer. Gold: Fe. Blue: N. Yellow: S.

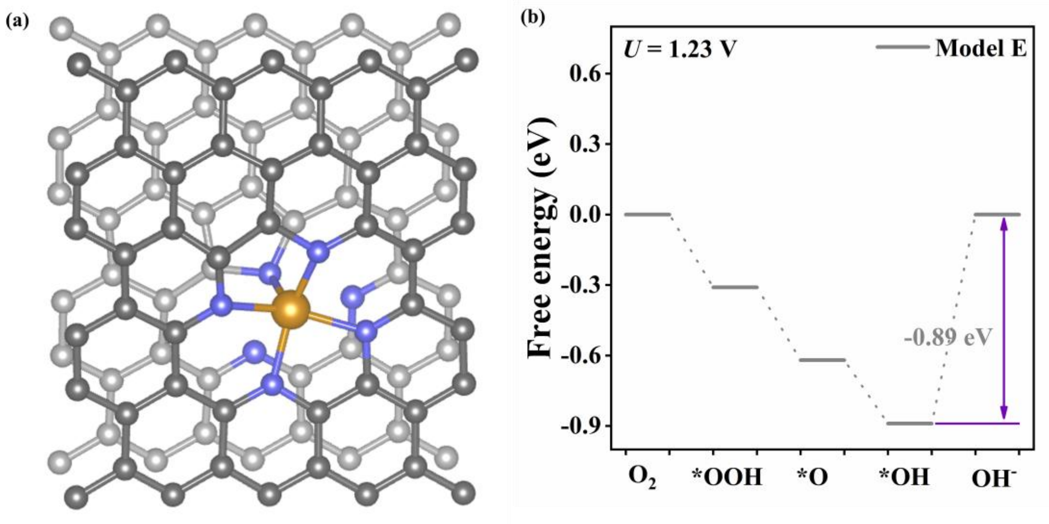

Figure S30 (a) Illustration of Model E (without S doping and micropores) and (b) free energy diagram of Model E at U = 1.23 V.

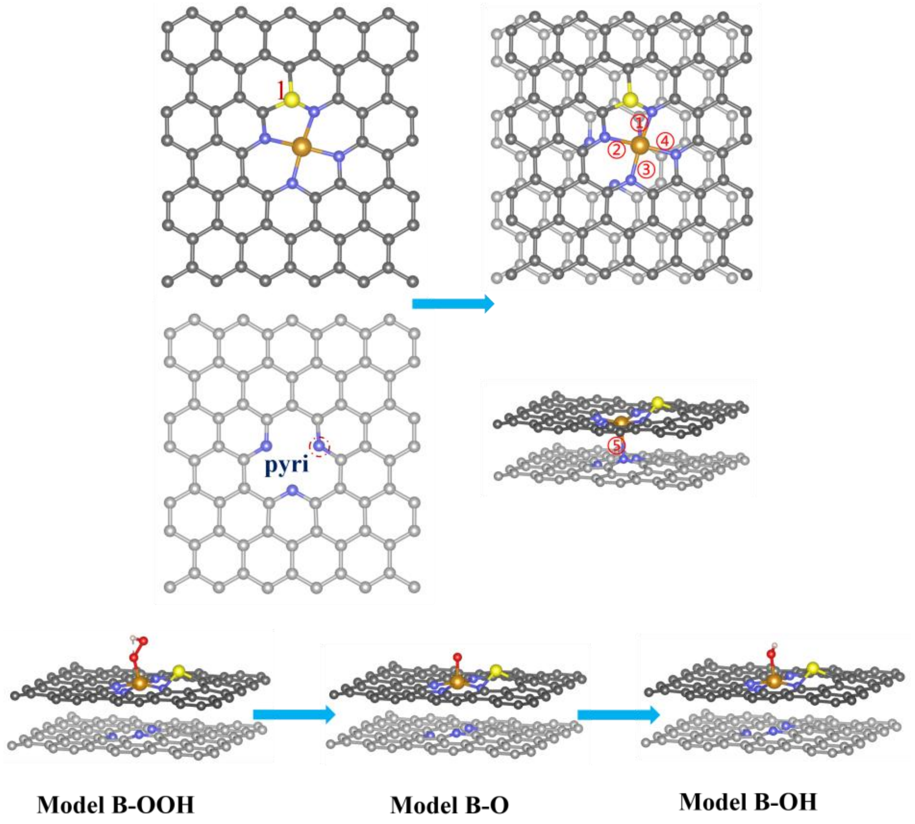

Figure S31 The structure of Model B and ORR reaction intermediates.

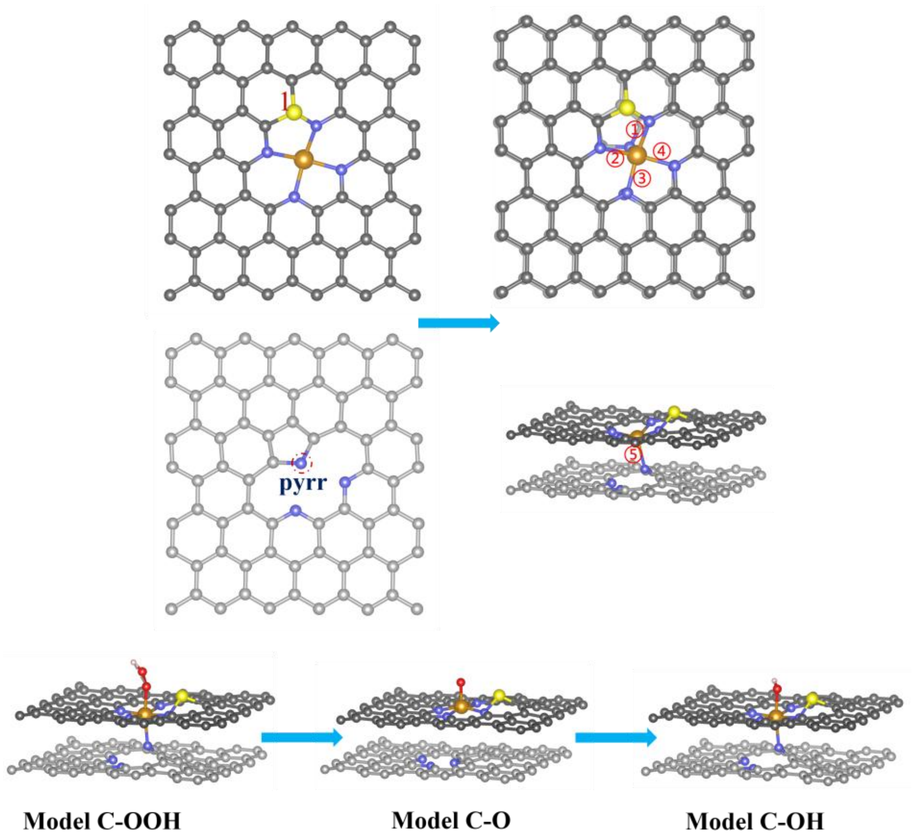

Figure S32 The structure of Model C and ORR reaction intermediates.

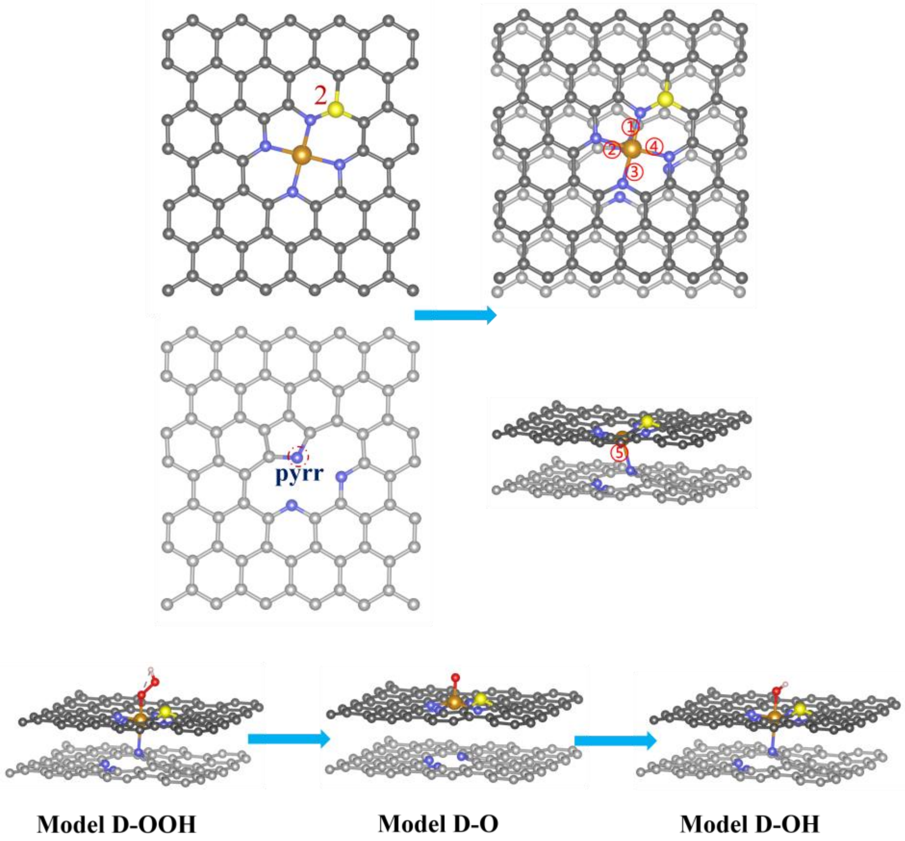

Figure S33 The structure of Model D and ORR reaction intermediates.

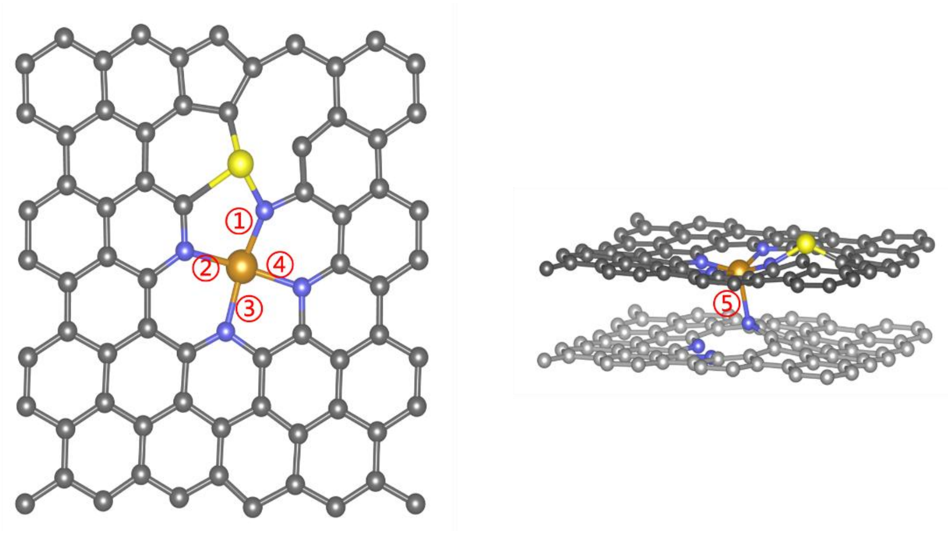

Figure S34 The naming order of the five Fe–N bonds in Fe–N_5.

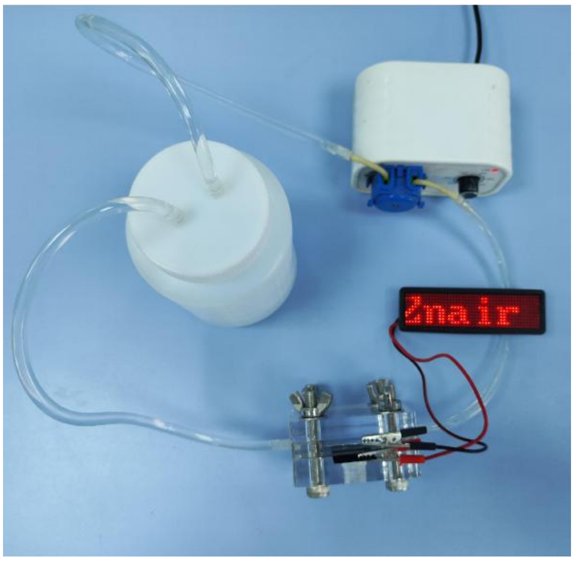

Figure S35 Photograph of the Zn-air battery.

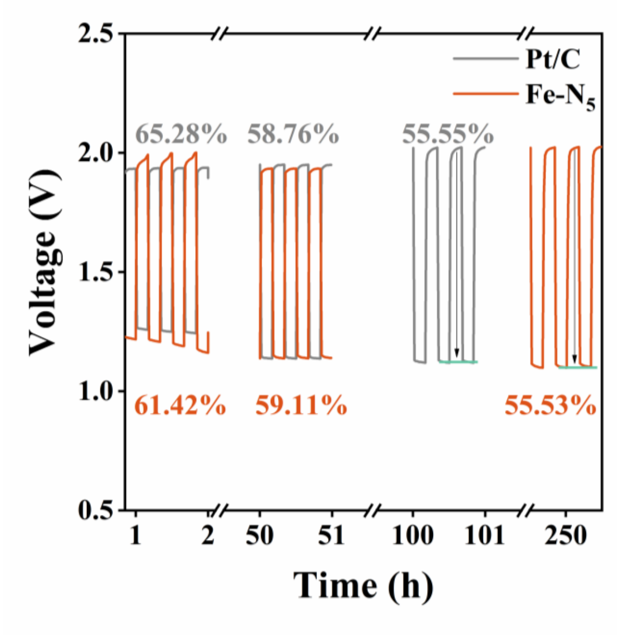

Figure S36 Round-trip efficiency graphs of Fe–N_5 + RuO_2 at 50, 250 h during the cycling test. (Time constraint = 20 min per cycle).

Table S1 The mass fraction (%) of S and Fe in Fe–N_5, Fe–N(100), Fe@H80, Fe@H100, Fe@CW, and Fe@W tested by ICP-MS.

Sample	S	Fe
Fe–N_5	0.090	0.61
Fe–N(100)	0.008	0.85
Fe@H80	0.850	0.45
Fe@H100	0.020	0.51
Fe@CW	1.780	0.33
Fe@W	-	0.27

Table S2 Mass Activity of Fe–N_5, Fe@CW, Fe@W, and Pt/C.

Sample	MA × 10^4 (A g_{Metal}^{-1})	
	0.85 V	0.90 V
Fe–N_5	2.4000	1.400
Fe@CW	2.1700	0.7500
Fe@W	1.8900	0.6700
Pt/C	0.0130	0.0054

Table S3 TOF values of $Fe–N_5$, Fe@CW, Fe@W, and Pt/C.

Sample	TOF (s^{-1})	
	0.85 V	0.90 V
$Fe–N_5$	3.680	2.040
Fe@CW	3.150	1.090
Fe@W	2.750	0.980
Pt/C	0.053	0.022

Table S4 Summary of the Q_{strip}, SD and TOF values of Fe–N_5, Fe@CW, and Fe@W.

Sample	Q_{strip} (C g^{-1})	SD (sties g^{-1})	TOF (e^- $site^{-1}$ s^{-1})
Fe–N_5	11.37	1.42×10^{19}	3.04
Fe@CW	6.27	7.84×10^{18}	1.11
Fe@W	9.02	1.12×10^{19}	0.71

Table S5 Structural parameters of samples obtained by fitting the EXAFS data.

sample	Scattering path	CN	R (Å)	E_0 (eV)	σ^2 ($Å^2$)	R factor
Fe foil	Fe–Fe1	8*	2.46	-2.59	0.0047	0.007
	Fe–Fe2	6*	2.84		0.0069	
FePc	Fe–N	3.9±0.1	1.94	-1.35	0.0089	0.014
$Fe–N_5$	Fe–N	5.3±0.2	2.03	2.29	0.0124	0.010

CN, coordination number; R, distance between absorber and backscatter atoms; σ^2, Debye-Waller factor to account for both thermal and structural disorders; ΔE_0, inner potential correction; R factor indicates the goodness of the fit. S_0^2 was fixed to 0.90. Fitting range: Δk = 2.5–12 $Å^{-1}$, ΔR =1–2 Å.

Table S6 Free energy for each reaction step at *U* = 1.23 V.

Sample	O_2	*OOH	*O	*OH	OH^-
Model A	0	-0.45 eV	-1.04 eV	-0.96 eV	0
Model B	0	-0.20 eV	-0.88 eV	-0.76 eV	0
Model C	0	0.21 eV	-0.82 eV	-0.42 eV	0
Model D	0	-0.16 eV	-0.60 eV	-0.73 eV	0
Model E	0	-0.31 eV	-0.62 eV	-0.89 eV	0
Fe–N_5	0	0.29 eV	-0.38 eV	-0.31 eV	0

Table S7 Free energy for each reaction step at $U = 0$ V.

Sample	O_2	*OOH	*O/H_2O_2	*OH	OH^-
Model A	4.92 eV	3.24 eV	1.42 eV	0.27 eV	0
Model B	4.92 eV	3.49 eV	1.58 eV	0.47 eV	0
Model C	4.92 eV	3.90 eV	1.64 eV	0.81 eV	0
Model D	4.92 eV	3.53 eV	1.86 eV	0.50 eV	0
Fe–N_5	4.92 eV	3.98 eV	3.37 eV		
Fe–N_5	4.92 eV	3.98 eV	2.08 eV	0.92 eV	0

Table S8 Fe–N bond lengths of reaction intermediates for Model B, Model C, Model D, and Fe–N_5.

sample	Fe–Nx	bond length
Model B	Fe–N1	1.90 Å
	Fe–N2	1.93 Å
	Fe–N3	1.90 Å
	Fe–N4	1.94 Å
	Fe–N5	2.40 Å
Model C	Fe–N1	1.92 Å
	Fe–N2	2.03 Å
	Fe–N3	1.96 Å
	Fe–N4	1.98 Å
	Fe–N5	2.16 Å
Model D	Fe–N1	1.88 Å
	Fe–N2	1.95 Å
	Fe–N3	1.92 Å
	Fe–N4	2.00 Å
	Fe–N5	2.21 Å
Fe–N_5	Fe–N1	1.86 Å
	Fe–N2	1.97 Å
	Fe–N3	1.97 Å
	Fe–N4	1.99 Å
	Fe–N5	2.12 Å

Table S9 Comparison of ORR performance in the alkaline media between prepared catalysts and the reported state-of-the-art catalysts in literatures.

Sample	E_{onset} (V vs.RHE)	$E_{1/2}$ (V vs.RHE)	J_k (mA cm^{-2})	Tafel slope (mV dec^{-1})	Ref.
Fe–N_5	1.11	0.964	6.90@0.95V<br>21.46@0.90V	61.9	This work
Fe SAs–HP	1.06	0.94	28.2@0.90V	84.0	[3]
Pt = N_2 = Fe ABA	1.050	0.950	5.83@0.95V<br>105.5@0.85V	58	[4]
PtFeNC	1.05	0.895	16.25@0.85V	-	[5]
Fe–N/P–C–700	0.941	0.867	24.49@0.85V	-	[6]
Fe,Mn/N–C	0.979	0.928	7.04@0.93V	79	[7]
Fe–SA–NSFC	1.01	0.91	61.5@0.85V	53	[8]
Fe SAs–Fe_2PNPs /NPCFs–2.5	1.03	0.91	14.47@0.85V	45.4	[9]
Fe SAs/NSC–vd	1.06	0.92	32.7@0.87V	75.2	[10]
Co_2/Fe–N@CHC	1.03	0.915	9.44@0.9V	62	[11]
Fe,P–DAS@MPC	1.02	0.92	-	49.8	[12]
Fe–N–DCSs	1.00	0.90	34.68@0.85V	62.8	[13]
Fe–SA/PNC	1.045	0.921	-	68	[14]
Fe–Se/NC	1.05	0.925	14.58@0.9V	56	[15]
NCAG/FeCu	1.07	0.94	25.5@0.85V	55	[16]

Supplementary References

[1] L. Jiao, J. Li, L. L. Richard, Q. Sun, T. Stracensky, E. Liu, M. T. Sougrati, Z. Zhao, F. Yang, S. Zhong, H. Xu, S. Mukerjee, Y. Huang, D. A. Cullen, J. H. Park, M. Ferrandon, D. J. Myers, F. Jaouen, Q. Jia, Nature Materials 2021, 20, 1385.

[2] Y. Liu, J. Li, Z. Lv, H. Fan, F. Dong, C. Wang, X. Chen, R. Liu, C. Tian, X. Feng, W. Yang, B. Wang, Journal of the American Chemical Society 2024, 146, 12636.

[3] P. Zhang, H.-C. Chen, H. Zhu, K. Chen, T. Li, Y. Zhao, J. Li, R. Hu, S. Huang, W. Zhu, Y. Liu, Y. Pan, Nature Communications 2024, 15.

[4] W. Zhou, H. Su, W. Cheng, Y. Li, J. Jiang, M. Liu, F. Yu, W. Wang, S. Wei, Q. Liu, Nature Communications 2022, 13.

[5] X. Zhong, S. Ye, J. Tang, Y. Zhu, D. Wu, M. Gu, H. Pan, B. Xu, Applied Catalysis B: Environmental 2021, 286.

[6] K. Yuan, D. Lützenkirchen-Hecht, L. Li, L. Shuai, Y. Li, R. Cao, M. Qiu, X. Zhuang, M. K. H. Leung, Y. Chen, U. Scherf, Journal of the American Chemical Society 2020, 142, 2404.

[7] G. Yang, J. Zhu, P. Yuan, Y. Hu, G. Qu, B.-A. Lu, X. Xue, H. Yin, W. Cheng, J. Cheng, W. Xu, J. Li, J. Hu, S. Mu, J.-N. Zhang, Nature Communications 2021, 12.

[8] Y. Zhou, X. Tao, G. Chen, R. Lu, D. Wang, M.-X. Chen, E. Jin, J. Yang, H.-W. Liang, Y. Zhao, X. Feng, A. Narita, K. Müllen, Nature Communications 2020, 11.

[9] Y. Pan, X. Ma, M. Wang, X. Yang, S. Liu, H. C. Chen, Z. Zhuang, Y. Zhang, W. C. Cheong, C. Zhang, X. Cao, R. Shen, Q. Xu, W. Zhu, Y. Liu, X. Wang, X. Zhang, W. Yan, J. Li, H. M. Chen, C. Chen, Y. Li, Advanced Materials 2022, 34.

[10] Y. Zhao, H. C. Chen, X. Ma, J. Li, Q. Yuan, P. Zhang, M. Wang, J. Li, M. Li, S. Wang, H. Guo, R. Hu, K. H. Tu, W. Zhu, X. Li, X. Yang, Y. Pan, Advanced Materials 2023, 36.

[11] Z. Wang, X. Jin, C. Zhu, Y. Liu, H. Tan, R. Ku, Y. Zhang, L. Zhou, Z. Liu, S. J. Hwang, H. J. Fan, Advanced Materials 2021, 33.

[12] L. Zong, K. Fan, P. Li, F. Lu, B. Li, L. Wang, Advanced Energy Materials 2022, 13.

[13] Y. Liu, L. Zong, Y. Zhang, F. Lu, L. Wang, Applied Catalysis B: Environment and Energy 2025, 361.

[14] W. Xue, Q. Zhou, X. Cui, J. Zhang, S. Zuo, F. Mo, J. Jiang, X. Zhu, Z. Lin, Angewandte Chemie International Edition 2023, 62.

[15] Y. Wang, J. Wu, S. Tang, J. Yang, C. Ye, J. Chen, Y. Lei, D. Wang, Angewandte Chemie International Edition 2023, 62.

[16] T. He, Y. Chen, Q. Liu, B. Lu, X. Song, H. Liu, M. Liu, Y. N. Liu, Y. Zhang, X. Ouyang, S. Chen, Angewandte Chemie International Edition 2022, 61.
